# Telomere-to-telomere haplotype-resolved reference genome reveals subgenome divergence and disease resistance in triploid Cavendish banana

**DOI:** 10.1093/hr/uhad153

**Published:** 2023-08-01

**Authors:** Hui-Run Huang, Xin Liu, Rida Arshad, Xu Wang, Wei-Ming Li, Yongfeng Zhou, Xue-Jun Ge

**Affiliations:** Key Laboratory of Plant Resources Conservation and Sustainable Utilization, South China Botanical Garden, Chinese Academy of Sciences, Guangzhou 510650, China; South China National Botanical Garden, Guangzhou 510650, China; Key Laboratory of Plant Resources Conservation and Sustainable Utilization, South China Botanical Garden, Chinese Academy of Sciences, Guangzhou 510650, China; South China National Botanical Garden, Guangzhou 510650, China; University of Chinese Academy of Sciences, Beijing 100049, China; State Key Laboratory of Tropical Crop Breeding, Shenzhen Branch, Guangdong Laboratory of Lingnan Modern Agriculture, Key Laboratory of Synthetic Biology, Ministry of Agriculture and Rural Affairs, Agricultural Genomics Institute at Shenzhen, Chinese Academy of Agricultural Sciences, Shenzhen 518120, China; State Key Laboratory of Tropical Crop Breeding, Shenzhen Branch, Guangdong Laboratory of Lingnan Modern Agriculture, Key Laboratory of Synthetic Biology, Ministry of Agriculture and Rural Affairs, Agricultural Genomics Institute at Shenzhen, Chinese Academy of Agricultural Sciences, Shenzhen 518120, China; School of Marine Sciences and Biotechnology, Guangxi University for Nationalities, Nanning 530008, China; State Key Laboratory of Tropical Crop Breeding, Shenzhen Branch, Guangdong Laboratory of Lingnan Modern Agriculture, Key Laboratory of Synthetic Biology, Ministry of Agriculture and Rural Affairs, Agricultural Genomics Institute at Shenzhen, Chinese Academy of Agricultural Sciences, Shenzhen 518120, China; State Key Laboratory of Tropical Crop Breeding, Tropical Crops Genetic Resources Institute, Chinese Academy of Tropical Agricultural Sciences, Haikou 571101, China; Key Laboratory of Plant Resources Conservation and Sustainable Utilization, South China Botanical Garden, Chinese Academy of Sciences, Guangzhou 510650, China; South China National Botanical Garden, Guangzhou 510650, China

## Abstract

Banana is one of the most important crops of the world. Cavendish-type bananas, which have a monospecific *Musa acuminata* origin (AAA), account for around half of the global banana production, thereby are of great significance for human societies. However, until now, the high-quality haplotype-resolved reference genome was still undecoded for banana cultivars. Here, we reported the telomere-to-telomere (T2T) and haplotype-resolved reference genome of ‘Baxijiao’ (Cavendish) consisting of three haploid assemblies. The sizes of the three haploid assemblies were estimated to be 477.16 Mb, 477.18 Mb, and 469.57 Mb, respectively. Although with monospecific origins, the three haploid assemblies showed great differences with low levels of sequence collinearity. Several large reciprocal translocations were identified among chromosomes 1, 4, and 7. An expansion of gene families that might affect fruit quality and aroma was detected, such as those belonging to sucrose/disaccharide/oligosaccharide catabolic processes, sucrose metabolic process, starch metabolic process, and aromatic compound biosynthetic process. Besides, an expansion of gene families related to anther and pollen development was observed, which could be associated with parthenocarpy and sterility of the Cavendish cultivar. Finally, much fewer resistance genes were identified in ‘Baxijiao’ than in *M. acuminata*, particularly in the gene clusters in chromosomes 3 and 10, providing potential targets to explore for molecular analysis of disease resistance in banana. This T2T haplotype-resolved reference genome will thus be a valuable genetic resource for biological studies, molecular breeding, and genetic improvement of banana.

## Introduction

Banana originated in Southeast Asia and the west Oceania region, where it was domesticated at least 7000 years ago [1]. Most banana cultivars are unseeded clones, mainly diploids or triploids, derived from wild fertile diploid *Musa* species. The genetic diversity of banana cultivars mostly resulted from hybridization events within *Musa acuminata* (A genome, 2n = 22), and between this species and *Musa balbisiana* (B genome, 2n = 22); meanwhile, to a minor extent, *Musa schizocarpa* (S genome, 2n = 22) and the species of *Australimusa* section (T genome, 2n = 22) also contributed to the genetic contents of edible banana cultivars [2]. Banana cultivars are among the most consumed fruits worldwide: over 100 million tonnes of banana are produced yearly [3]. Besides, banana cultivars are also the fifth most produced food crop in the least-developed countries; as a result, they play an important role in maintaining food security in these regions. The demand for cultivated bananas continues to grow; however, their production is greatly affected by a complex of biotic and abiotic stresses. For instance, banana yields are severely threatened by the disease fusarium wilt, which is caused by the fungus *Fusarium oxysporum* f. sp. *cubense* (*Foc*) [4]. Therefore, breeding new banana cultivars with improved traits, particularly genetic resistance to a wide and diverse group of pathogens, is crucial for future banana production; and a high-quality banana genome would no doubt facilitate such genetic manipulation.

Efforts to sequence banana genomes began over 10 years ago. D’Hont *et al.* [5] reported the first draft genome of a doubled-haploid Pahang (DH-Pahang) *M. acuminata* genotype of the subspecies *malaccensis*, providing a key stepping-stone for genetic breeding for banana. Since then, the DH-Pahang genome has been improved twice [6, 7]. Moreover, other *Musa* genomes, for instance *M. balbisiana*, *Musa itinerans*, *M. schizocarpa*, *Musa troglodytarum*, and *Musa beccarii*, have already been reported during the past decade [2, 8–12]. These high-quality banana genomes have provided necessary and valuable resources for further research on evolution of *Musa* genus, as well as domestication and breeding of banana. Technological advances have boosted sequencing capacities and made the sequencing of complex genomes from multiple species and cultivars available. Thanks to long-read sequencing, now telomere-to-telomere (T2T) gap-free assembly of chromosome sequences is possible, providing unprecedented opportunities to untangle genomic regions missed in previous assemblies [13]. However, there has long been a lack of high-quality genomes for banana cultivars, likely due to the complexity of the genomes [14–16].

In banana, the most efficient ploidy level for agronomic performance is triploid, which has given rise to more vigorous plants, higher sterility, and larger fruits without seeds. Cavendish-type bananas, which have a monospecific *M. acuminata* origin (AAA), account for around half of the global banana production [1, 17]. Nearly all of the global banana export trade relies on the clones derived from a unique triploid cultivar of Cavendish banana [17], leading to a narrow genetic basis in this type of banana. In this study, we developed a T2T reference genome of a banana cultivar ‘Baxijiao’ (AAA, Cavendish) consisting of three haploid assemblies, by integrating PacBio high-fidelity (HiFi) reads, Oxford Nanopore Technology (ONT) ultra-long reads, and Hi-C reads. This haplotype-resolved reference genome revealed great differences among the three haploid assemblies with low levels of sequence collinearity. In addition, some enriched biological processes of ‘Baxijiao’ specific gene families were found to be related to fruit quality and aroma, as well as parthenocarpy and sterility of the cultivar. Finally, the presence of much fewer resistance genes in the ‘Baxijiao’ genome than in *M. acuminata* genome shed light on the possible mechanisms of fusarium wilt susceptibility of this cultivar. We hope this high-quality genome will help to obtain a better understanding of banana domestication, and open a new range of opportunities for biological studies and the breeding of banana varieties.

## Results

### Assembly of highly contiguous and phased triploid reference genome

We generated the ‘Baxijiao’ genome by incorporating PacBio HiFi sequences, Oxford nanopore sequences, and Hi-C sequences. In total, we obtained 102.06 Gb (~72× coverages of the total genome size) of PacBio HiFi reads with N50 longer than 15 kb, 48.00 Gb (~34× coverages) of ONT reads with N50 of 80 kb, and 156.14 Gb (~110× coverages) of Hi-C data, respectively (Table S1, see online supplementary material).

**Figure 1:**
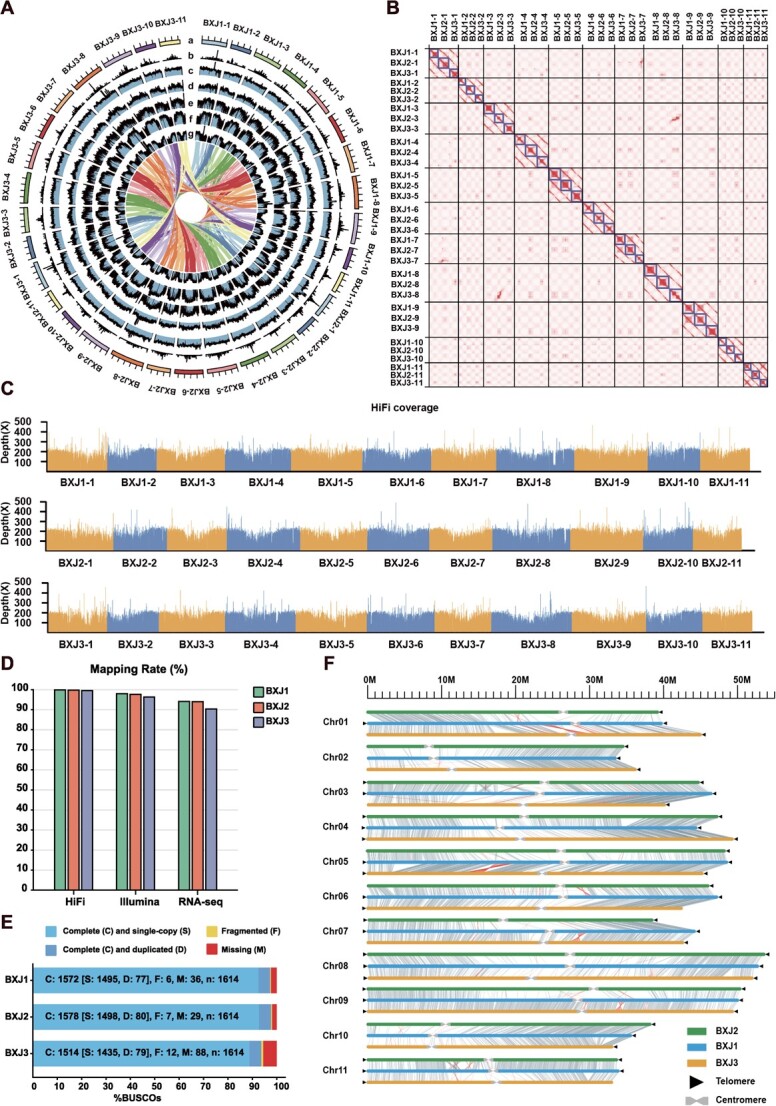
Overview of the Cavendish reference genome. **A** The circos diagram of the ‘Baxijiao’ genome. The tracks represent the following elements (from outer to inner): (a) schematic representation of the 33 chromosome sequences, (b) density of centromeric repeats, (c) GC contents, (d) density of transposable elements, (e) density of the Gypsy elements, (f) density of the Copia elements, (g) density of genes. The innermost is syntenic relationships. **B** Hi-C interaction heatmap for the ‘Baxijiao’ genome. **C** HiFi coverage of the 33 pseudo-chromosomes. **D** Mapping rates of HiFi, Illumina and RNAseq reads in BXJ1, BXJ2, and BXJ3. **E** BUSCO assessments in BXJ1, BXJ2, and BXJ3. **F** Sequence collinearity among BXJ1, BXJ2, and BXJ3. Gray lines represent the collinearity blocks with length of 15 000 bp, while red lines represent the potential inversions.

The haploid genome of ‘Baxijiao’ was estimated to be ~457.31 Mb with a heterozygous rate of 2.855% by GenomeScope 2.0 [18] based on a k-mer of 21 (Fig. S1, see online supplementary material). To achieve the T2T and haplotype-resolved genome, we adopted an assembly scheme that allows combining HiFi and Hi-C reads to assemble the phased genome coupled with ONT reads to fill the gaps (Fig. S2, see online supplementary material). Based on HiFi and Hi-C reads, hifiasm [19] was applied to build a phased diploid assembly, generating 581.77 Mb and 959.85 Mb primary contigs with N50 of 14.95 Mb and 16.15 Mb, respectively. With the aid of the T2T genome of *M. acuminata* ssp. *malaccensis* (X. Liu *et al.* unpublished results), the contigs were further ordered, orientated, and grouped. Then Hi-C reads were employed to anchor and remove some short contigs, in addition to splitting the larger contigs into two sets. NextDenovo (https://github.com/Nextomics/NextDenovo) was used to construct the ONT assembly, which was subsequently used to fill the gaps of the HiFi-assembled reference. After filling gaps, a nearly complete and phased reference genome named BXJ was generated, containing 33 pseudo-chromosomes (11 for each haploid assembly) with a total length of 1.42 Gb, and sizes of 469.57–477.18 Mb for haploid assemblies (Fig. 1A andTable 1; Table S2, see online supplementary material). The three haploid assemblies were named BXJ1, BXJ2, and BXJ3. The longest chromosome was chromosome 8 for each haploid assembly, with a size of 53.00 Mb, 54.63 Mb, and 52.65 Mb for BXJ1, BXJ2, and BXJ3, respectively. The shortest chromosome showed slightly different between BXJ1 and the other two haploid assemblies; specifically, chromosome 2 was the shortest chromosome in BXJ1 (33.52 Mb), while chromosome 11 was the shortest one in both BXJ2 (33.79 Mb) and BXJ3 (32.90 Mb). Using 100-kb intervals, the gene density, TE density, *Copia* density, *Gypsy* density, GC contents and potential centromere tandem repeats density were counted in the BXJ genome, respectively (Fig. 1A). This is the first T2T and haplotype-resolved reference genome for triploid bananas.

**Table 1:** Genome assembly statistics for ‘Baxijiao’ and MAv4

	**Baxijiao**			**MAv4**
	BXJ1	BXJ1	BXJ1	
Cumulative size (bp)	477 162 536	477 181 898	469 568 689	468 821 802
HiC scaffolds	11	11	11	11
Complete BUSCO (N = 1614)	97.40%	97.80%	93.80%	98.80%
Gaps	33	40	71	15
After gap-closing	0	2	0	15
Number of Telomere	19	17	17	22
HiFi mapping rate	99.84%	99.80%	99.50%	
Illumina mapping rate	97.95%	97.66%	96.36%	
Repeats	53.76%	54.14%	55.13%	52.62%
LAI index	19.84	20.65	20.22	
Number of predicted genes	37 185	37 241	37 178	36 979

### Genome annotation and quality assessment

Within the three haploid assemblies, 256.48 Mb (53.76%), 258.29 Mb (54.14%), and 258.84 Mb (55.13%) of repetitive regions were identified (Table S3, see online supplementary material), similar to those in other banana genomes (e.g. *M. acuminata*: 52.62% [7]; *M. beccarii*: 55.99% [12]). Among them, the most abundant repetitive sequences were long terminal repeats (LTRs), which accounted for 36.36%, 35.44%, and 33.53% of the BXJ1, BXJ2, and BXJ3 assemblies, respectively (Table S3, see online supplementary material). A total of 37 185, 37 241, and 37 178 high-confidence protein-coding genes were predicted from BXJ1, BXJ2, and BXJ3 (Table 1), which were slightly more than the genes (36 979) predicted in the latest v4 version of the *M. acuminata* double-haploid genome (hereafter MAv4) [7]. The total lengths of the predicted genes in BXJ1, BXJ2, and BXJ3 were 142.78 Mb, 138.99 Mb, and 133.28 Mb, respectively. The average lengths of these protein-coding genes were 3 839 bp, 3 732 bp, and 3 584 bp.

Using the seven-base repeats (CCCATTT at the 5′ end and TTTAGGG at the 3′ end) as queries, we totally estimated 53 telomeres and constructed 8, 6, and 6 telomere-to-telomere chromosomes for BXJ1, BXJ2, and BXJ3, respectively (Table S4, see online supplementary material). The length of the identified telomeres ranged from 6 006 to 33 838 bp in BXJ1, 2 345 to 44 905 bp in BXJ2, and 4 494 to 26 964 bp in BXJ3. The longest telomere sequence was located at the right end of Chr10 in BXJ2, whereas the shortest one was located at the right end of Chr08 in BXJ2.

Centromere is a specialized nucleotide sequence in a chromosome that holds together the two daughter chromatids and is involved with the movement in mitosis and meiosis. Using Tandem Repeats Finder [20], the approximate locations of the centromeres in the ‘Baxijiao’ genome were identified, with lengths ranging from 924 207 to 3 415 439 in BXJ1, 930 592 to 3 844 332 in BXJ2, and 481 633 to 4 080 464 bp in BXJ3, respectively (Table S5 and Fig. S3, see online supplementary material).

The quality and completeness of the BXJ assembly were evaluated in multiple ways. First, as estimated, the genome sizes of each haploid assembly (469.57–477.18 Mb) were slightly longer than that of MAv4 (~468.82 Mb) [7]; in addition, Hi-C data visualized by JuiceBox [21] presented a high consistency across all chromosomes, providing their accuracy of the ordering and orientation (Fig. 1B). Second, both HiFi and Illumina reads were aligned to the three haploid assemblies; the average HiFi read coverages of each chromosome were ~200 × (Fig. 1C); besides, 97.95%, 97.66%, and 96.36% Illumina reads, as well as 99.84%, 99.80%, and 99.50% HiFi reads were successfully aligned to BXJ1, BXJ2, and BXJ3, respectively (Fig. 1D), indicating sufficient and high coverage of the ‘Baxijiao’ genome by this assembly. Third, high LTR assembly index (LAI) scores (19.84, 20.65, and 20.22) were observed in the three haploid assemblies (Table 1). Finally, evaluation of completeness using BUSCO [22] revealed that complete sequences of BXJ1, BXJ2, and BXJ3 accounted for 97.40%, 97.80%, and 93.80% of the conserved core eukaryotic gene set, respectively (Table 1 and Fig. 1E). Overall, these results presented the high quality, accuracy, and reliability of the BXJ reference genome.

### Whole genome comparison and syntenic analysis

**Figure 2:**
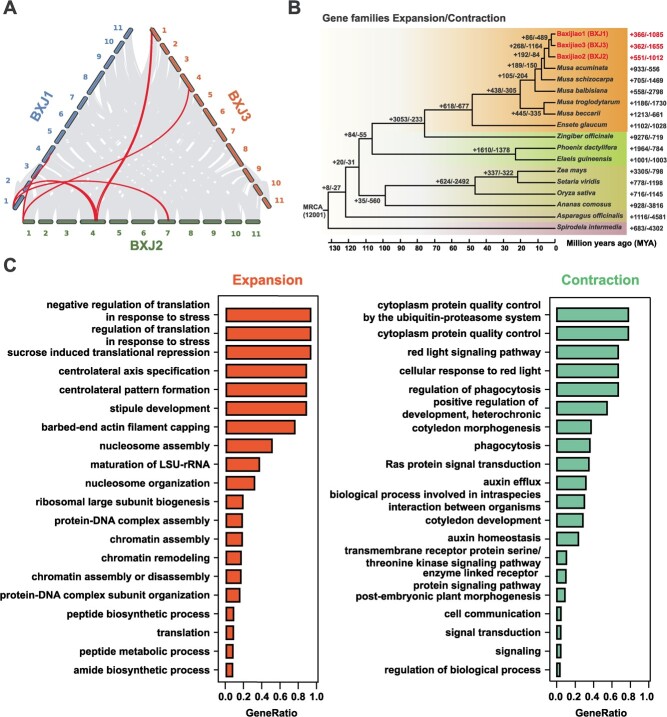
Whole genome comparisons within ‘Baxijiao’ and between ‘Baxijiao’ and other species in monocots. **A** Syntenic analysis at the gene level. Highlighted lines represent reciprocal translocations. The other lines represent syntenic blocks. **B** Inferred phylogenetic tree and the expanded (+) or contracted (−) gene families in ‘Baxijiao’ and other species in monocots. Number of expanded and contracted gene families at branch (share between species/BXJ haploid assemblies) and leaf nodes (share in specific species/BXJ haploid assemblies) has been denoted. **C** The top enriched biological processes for expanded and contracted genes in ‘Baxijiao’.

BXJ1, BXJ2, and BXJ3 harbor a set of similar genomic features, such as similar genome sizes and gene numbers, as well as parallel repeat contents (Table 1). However, the syntenic analysis revealed low levels of nucleotide sequence collinearity between the haploid assemblies (Fig. 1F), which could be attributed to the different origins of *M. acuminata* subspecies of the subgenomes. Meanwhile, due to evolutionary conserved coding regions, high syntenic relationships of genes among the three haploid assemblies were observed. Genomic collinearity analysis revealed that 29 131, 27 571, and 28 838 transcripts matched 31, 37, and 37 syntenic blocks in the three pairwise comparisons (BXJ1 vs BXJ2, BXJ1 vs BXJ3, BXJ2 vs BXJ3), respectively. Substantial structural variations within the BXJ genome were identified using MCScan [23]. Several large reciprocal translocations were identified among Chr01, Chr04, and Chr07 (Fig. 2A). Specifically, syntenic relationships of Chr01 and Chr04 were observed, indicating the occurrence of a reciprocal translocation involving the distal end of the long arm of Chr01 in BXJ2 and a 0.47–0.50 Mb region in the long arm of Chr04 in BXJ1 and BXJ3 (Fig. S4A, see online supplementary material). In addition, another reciprocal translocation involving a 1.88–3.12 Mb region in the distal end of the long arm of Chr01 in BXJ1 and BXJ3 and the long arm of Chr04 in BXJ2 was also identified (Fig. S4B, see online supplementary material). Moreover, syntenic relationship of Chr01 and Chr07 was also observed, showing the existence of a reciprocal translocation involving the distal end of the long arm of Chr01 in BXJ1 and a 1.04 Mb region in Chr07 in BXJ2 (Fig. S4C, see online supplementary material).

According to sequence identity, we obtained 18 263 groups of alleles between the haplotype-resolved BXJ genome and MAv4 (Table S6, see online supplementary material). These alleles are evenly distributed across the 11 chromosomes of the genome. The coding sequences of most allelic genes in the three haploid assemblies were highly similar to those in MAv4 (mean = 96.50%, 96.59%, and 96.62%). In addition, through calculating Ka/Ks values between the allelic gene pairs, we found that most allelic genes had experienced purifying selection (Ka/Ks < 1, Fig. S5, see online supplementary material).

### Gene family evolution

Phylogenomic analysis was performed using the homologous genes from ‘Baxijiao’ and 15 other species in monocots, including the main ancestral contributors of banana cultivars: *M. acuminata* (A genome), *M. balbisiana* (B genome), *M. schizocarpa* (S genome), and *M. troglodytarum* (T genome). The phylogenetic tree suggested that the three haploid assemblies of ‘Baxijiao’ were clustered together, and sister to *M. acuminata* (Fig. 2B).

In total, 38 387 orthogroups were characterized from a set of 612 509 genes from the chosen monocot plant species. Based on the phylogenetic tree, potential expanded and contracted gene families were identified. In total, 447 significant expansion and contraction gene families consisting of 1 551 genes were specific to ‘Baxijiao’. A total of 117 gene families composed of 694 genes have significantly expanded in the ‘Baxijiao’ genome. GO enrichment analysis revealed that these expanded genes were significantly enriched in 199 biological processes (Table S7, see online supplementary material). The most enriched biological processes were related to response to stress and sucrose induced translational repression (Fig. 2C). Interestingly, some other significantly enriched biological processes were associated with sources of carbohydrates and pollen development, including response to disaccharide, response to sucrose, response to carbohydrate, acceptance of pollen, recognition of pollen, pollen-pistil interaction, and pollen germination. Meanwhile, 330 gene families composed of 857 genes have significantly contracted, which were enriched in 231 biological processes (Table S8, see online supplementary material). A large proportion of the enriched biological processes (30%) were associated with plant growth, morphogenesis, and hormones, such as cotyledon development, fruit development, anther development, floral organ morphogenesis, developmental process involved in reproduction, auxin efflux, hormone-mediated signaling pathway, and so on. In addition, some of the enriched biological processes (14%) were associated with response to external biotic and abiotic factors, including response to symbiotic fungus, response to bacterium, immune response-activating signal transduction, red or far-red light signaling pathway, and cellular response to blue light. Potential expanded and contracted gene families were also characterized in the haploid assemblies (Table S9, see online supplementary material). Interestingly, some significantly expanded gene families were enriched in aromatic compound biosynthetic process, sucrose catabolic process, disaccharide catabolic process, oligosaccharide catabolic process, sucrose metabolic process, and starch metabolic process, which might be important for fruit quality (Fig. S6 and Tables S10–S12, see online supplementary material). In addition, some expanded gene families were involved in anther, particularly anther wall tapetum development, such as anther morphogenesis and anther wall tapetum formation.

### Resistance genes

**Figure 3:**
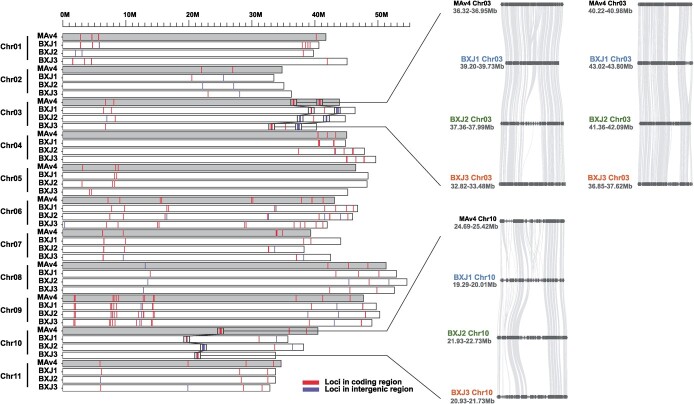
The distribution of NLR loci in MAv4 and ‘Baxijiao’ genomes. Lines on chromosomes suggest that the loci are located in coding regions and intergenic regions, respectively. The syntenic relationships of the gene clusters in chromosome 3 and 10 are presented in the right panel.

Plant genomes typically include a great number of disease resistance (R) genes. There are two main categories of plant R proteins: membrance-bound pattern recognition receptors (PRRs) and intracellular nucleotide-binding, leucine-rich repeat receptors (NLRs). PRRs are either receptor-like kinases (RLKs) or receptor-like proteins (RLPs) [24]. In this study, we first employed RGAugury [25] to predict resistance gene analogs (RGAs) in ‘Baxijiao’ and *M. acuminata*. A total of 68, 52, and 69 NBS-encoding genes were predicted in BXJ1, BXJ2, and BXJ3, respectively, compared to 124 in MAv4 (Table 2). The prediction for RLK- and RLP-type RGAs also suggested that ‘Baxijiao’ harbors considerably fewer R genes than *M. acuminata* (Table 2). The distribution of the above predicted RGAs in MAv4 and ‘Baxijiao’ genomes was presented in supplementary Fig. S7 (see online supplementary material). In addition, we applied the NLR-annotator [26] to characterize the genomic regions likely associated with disease resistance. An NLR locus predicted might be a gene with a complete or partial open reading frame but might also be the trace of a pseudogenized sequence in the investigated genome [26]. A total of 123, 126, and 140 NLR loci were detected in BXJ1, BXJ2, and BXJ3, respectively (Table 2; Tables S13–S15, see online supplementary material), compared to 128 loci in MAv4 [7]. NLR loci were unevenly distributed on all 11 chromosomes (Fig. 3). Some NLR loci were found to cluster together preferentially. Three major clusters of the predicted NLR loci were observed in the BXJ genome: two in Chr03 and one in Chr10 (Table 3). It is worth noting that a large proportion of NLR loci in the BXJ genome were found to be located in intergenic regions (BXJ1: 37.4%; BXJ2: 59.5%; BXJ3: 38.6%). Only 77, 51, and 79 resistance genes were predicted in the three haploid assemblies compared to 122 genes in MAv4, similar to the prediction for NBS-encoding RGAs. Especially in the gene clusters in the distal end of the long arm of Chr03 (BXJ1: 43.02–43.80 Mb; BXJ2: 41.36–42.09 Mb; BXJ3: 36.85–37.62 Mb), all NLR loci were predicted to be pseudogenized sequences (Fig. 3 and Table 3). In addition, in the gene cluster in Chr10, all NLR loci were predicted to be pseudogenized in BXJ2 (21.93–22.73 Mb), while most NLR loci in BXJ1 (19.29–20.01 Mb) and BXJ3 (20.93–21.73 Mb) remained to be functional according to gene annotation (Fig. 3 and Table 3). Overall, much fewer resistance genes were identified in ‘Baxijiao’ than in *M. acuminata*.

**Table 2:** Summary of the predictions by RGAugury and NLR-annotator

Genome	RGAugury			NLR-annotator	
	NBS encoding	RLK	RLP	NLR loci	NLR gene
MAv4	124	752	109	128	122
BXJ1	68	554	63	123	77
BXJ2	52	541	87	126	51
BXJ3	69	468	62	140	79

**Table 3:** Comparison of NLR clusters between the BXJ and MAv4 assemblies

**Chr**	**Location (Mb)**				**No. NLR genes / No. NLR loci**			
	BXJ1	BXJ2	BXJ3	MAv4	BXJ1	BXJ2	BXJ3	MAv4
Chr03	39.20–39.73	37.36–37.99	32.82–33.48	36.32–36.95	3/8	0/12	19/20	15/16
Chr03	43.02–43.80	41.36–42.09	36.85–37.62	40.22–40.98	0/26	0/17	0/33	14/18
Chr10	19.29–20.01	21.93–22.73	20.93–21.73	24.69–25.42	8/13	0/18	10/17	12/13

No. NLR genes: number of NLR genes predicted.

No. NLR loci: number of NLR loci detected.

### Homoeolog expression patterns in phased triploid reference genome

**Figure 4:**
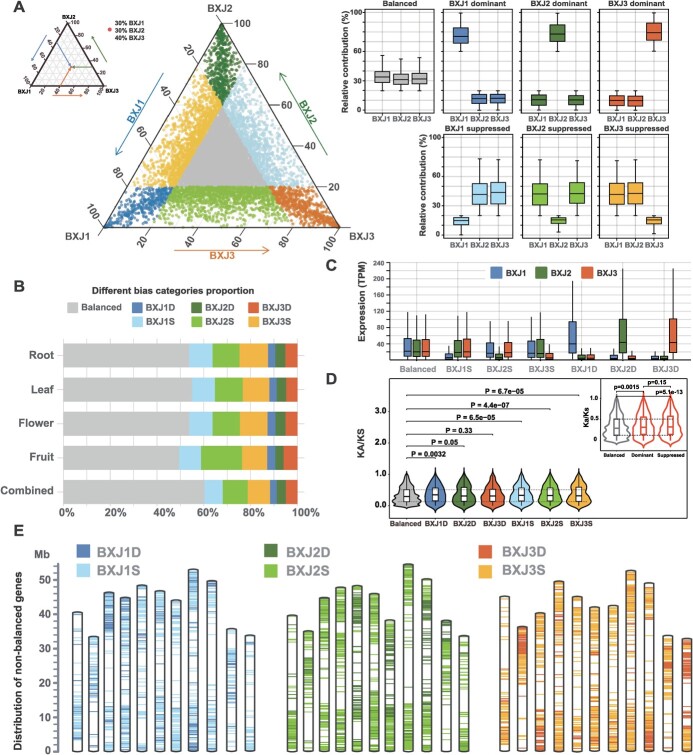
Homoeolog expression bias in syntenic homoeolog triads. **A** Ternary plot showing relative expression levels of 17 531 syntenic triads (52 593 genes) in ‘Baxijiao’. Each circle represents a gene triad with a BXJ1, BXJ2, and BXJ3 coordinate suggesting the relative contribution of each homoeolog to the overall triad expression (an example is shown on the top left). The gray circles represent the balanced triads, while the colored circles in vertices correspond to the triads from the homoeolog-dominant groups, and the colored circles close to edges and between vertices correspond to the triads from the homoeolog-suppressed groups. Box plots indicate the relative contribution of each homoeolog based on triad assignment to the seven groups, including BXJ1D, BXJ2D, BXJ3D representing BXJ1, BXJ2 and BXJ3 dominant respectively, and BXJ1S, BXJ2S and BXJ3S represent BXJ1, BXJ2, and BXJ3 suppressed. **B** Contribution of each group of homoeolog expression bias across the 4 tissues and in the combined analysis. **C** Box plot of absolute TPM expression abundance for each haploid genome from the seven groups. **D** Violin plots of the Ka/Ks values for the seven categories. The balance category shows slightly lower Ka/Ks values than the homoeolog-dominant and homoeolog-suppressed groups. **E** The distribution of homoeolog expression bias genes in all 33 chromosomes.

To determine patterns of homoeolog expression, we focused on 52 593 expressed genes (17 531 syntenic triads in total 18 263 triads, 95.99%) that had a 1:1:1 gene correspondence across the three haploid assemblies, and a homoeolog expression of >0.5 transcripts per million (TPM) in at least one tissue (Table S16, see online supplementary material). For each of the 52 593 expressed triads, we first standardized the relative expression of the BXJ1, BXJ2, and BXJ3 homoeologs for each individual tissue and combined analysis. We then carried out a global analysis integrating data across all four tissues (Fig. 4A). On average, ~40% of homoeolog triads showed non-balanced expression patterns, with higher (homoeolog-dominant) or lower expression (homoeolog-suppressed) from a single homoeolog respecting the other two. As for each tissue, balanced triads ranged from 49.45% in fruit to 55.00% in leaf (Fig. 4B). The homoeolog-dominant triads were found at moderate frequencies for each tissue (ranging from 11.95% to 12.83%), whereas the homoeolog-suppressed triads were more frequent (from 33.06% to 37.72%). Genes from syntenic triads in the dominant groups had higher absolute transcript abundance than genes in the balanced or suppressed groups (Fig. 4C). In addition, we found that the non-balanced triads showed subtly higher Ka/Ks values than the balanced triads (Fig. 4D). GO enrichments were carried out on the non-balanced triad; however, significant functional enrichment was not observed for these genes. The density of non-balanced expression genes was presented in Fig. 4E, which showed higher abundance of these genes along the regions near the ends of chromosomes.

## Discussion

Banana is among the most popular fruits and the fifth most produced food crop worldwide. The main traits selected in banana during domestication are parthenocarpy and sterility, which ensure the formation of fleshy and edible fruits without seeds. Genetic improvement and breeding for banana is challenging, because it is hard to generate high-quality parthenocarpic and sterile varieties through the recombination of non-parthenocarpic and fertile wild species. In this context, the genomic characterization of banana cultivars and a deep understanding of the wild-to-domesticate transition are crucial for successful banana breeding schemes. Here we assembled the T2T and haplotype-resolved reference genome of ‘Baxijiao’, a representative of the most commercialized Cavendish bananas. Our incorporated assembly scheme involving HiFi reads, ONT ultra-long reads, and Hi-C reads generated a high-quality BXJ genome, achieving the assembly goals of high contiguity and accuracy. Multiple evaluations including genome size and consistency, read mapping, LAI scores, and BUSCO supported the high quality, accuracy, and reliability of the BXJ assembly. Moreover, we identified the locations of the telomeres and centromeres in the chromosomes. To the best of our knowledge, this is the first T2T and phased reference genome for triploid bananas. This genome will thus provide valuable genetic resources for further research on banana domestication, and molecular breeding and genetic improvement of banana cultivars.

### The haplotype-resolved reference genome of triploid Cavendish cultivar showed sequence and expression divergences among subgenomes

The domestication process has left signatures in the genomes of domesticated species. Cultivated bananas were derived from inter- or intra-specific *Musa* hybrids [2]. Most banana cultivars were derived from *M. acuminata* (A genome), a complex of subspecies that are geographically segregated in distinct continents and islands in Southeast Asia [27]. Four specific *M. acuminata* subspecies have been raised as the main parental contributors of banana cultivars, namely *banksii*, *burmannica*, *malaccensis*, and *zebrina* [1]. Accumulating evidence has put forward the subspecies *banksii* from New Guinea, *zebrina* from Java, and *malaccensis* from Malaysia and Sumatra as the main contributors to the genetic makeup of banana cultivars, and the minor involvement of *burmannica* [28, 29]. In addition, several recent studies on banana cultivars with pure *M. acuminata* origins proposed the presence of missing ancestral gene pools involving the setup of banana cultivars [15, 16, 30]. Cavendish-type bananas have a monospecific *M. acuminata* origin (AAA). Previous studies have established the contribution of the ‘Mlali’ subgroup, of *banksii* and *zebrina*/*microcarpa* ascendance, as 2 N gamete donor to Cavendish bananas; these studies also suggested that the N gamete was delivered by an AA diploid with a *malaccensis* affiliation [27, 31]. However, the hybridization events during banana domestication were far more frequent than previously expected, leading to over three contributors to Cavendish bananas. It has been reported that Cavendish bananas harbored mixed mosaic profiles with genomic regions of *M. acuminata* subspecies *banksii*, *zebrina*, and *malaccensis*, along with *M. schizocarpa*, and two unknown contributors [16]. As for ‘Baxijiao’, there existed low levels of sequence collinearity between any two haploid assemblies, consistent with the complex inter- or intra-specific hybrid origin of Cavendish bananas. Furthermore, the chromosomal rearrangements, the expanded/contracted gene families, as well as the homoeolog expression patterns also exhibited differences among the three haploid assemblies, further supporting the complex story of hybridization in triploid bananas.

In this study, we detected an expansion of gene families related to various processes that might affect fruit quality in ‘Baxijiao’, such as sucrose/disaccharide/oligosaccharide catabolic processes, sucrose metabolic process, starch metabolic process, and aromatic compound biosynthetic process. Soluble sugars (e.g. sucrose, glucose, and fructose) have a significant impact on fruit flavor and taste. Soluble sugars also provide precursors for the synthesis of other compounds associated with fruit quality such as amino acids, organic acids, and antioxidant micronutrients. In general, the sugar content of many modern fruits changed to a great extent due to continuous selection and breeding efforts [32, 33]. The sugar contents are quite low at the early post-harvest stage of banana. During fruit development, banana accumulates a large reserve of starch (15–35% w/w of their fresh weight). However, due to a complex metabolism of starch shifting from synthesis to breakdown, the starch content at late ripening decreases drastically and the soluble sugars accumulate [34]. This transition from starch to sugar appears to be accountable both for pulp sweetening and for providing energy to metabolic processes that determine other quality characteristics of ripe banana; for instance, synthesis of volatile compounds, color change of fruit peel, and even pulp softening, thereby playing important roles in final fruit quality [35]. In addition, aromatic compounds are essential for fruit quality because the aroma is a major indicator of fruit flavor. Banana fruits have a pleasant flavor. Typical flavor compounds are synthesized during banana ripening, forming unique aroma signatures of banana fruits [36]. The aromatic profiles of many banana cultivars have been broadly uncovered, indicating that the characteristic banana aroma was the result of a complex mixture of volatile compounds [37–39]. In this sense, the expanded biological processes associated with carbohydrate metabolism and aromatic compound biosynthesis in the ‘Baxijiao’ genome might be a different feature from its wild ancestors formed in its domestication process and play important roles in its fruit quality. We also observed an expansion of gene families associated with anther and pollen development in the ‘Baxijiao’ genome. The cultivar ‘Baxijiao’ is triploid and parthenocarpic, thereby producing fruits that are free of seeds, while *M. acuminata* has fruits containing numerous seeds making them inedible [40]. Relatively, according to our observation, ‘Baxijiao’ has thin anthers without pollens, but *M. acuminata* harbors anthers with larger size and full of pollens. Therefore, these expanded gene families in the ‘Baxijiao’ genome could be responsible for the reproductive difference between cultivated ‘Baxijiao’ and its wild ancestors. The anther wall tapetum is a layer of cells that provides a source of nutrition for the pollen grains as they mature [41, 42]. Here the expanded gene families were enriched in several biological processes related to anther wall tapetum, which were expected to play crucial roles in the parthenocarpic characteristic of ‘Baxijiao’. Some contracted gene families in the ‘Baxijiao’ genome were involved in the biological processes associated with response to external biotic and abiotic factors. Accumulating evidence indicates that banana cultivars have generally reduced resistance to pathogens and environmental stresses than their wild ancestors [43, 44]. These contracted gene families will provide candidates for further investigations on the genetic basis of the reducing resistance in banana cultivars.

Here we also investigated the homoeolog expression patterns in ‘Baxijiao’. On average, ~40% of homoeolog triads presented non-balanced expression patterns with higher (homoeolog-dominant) or lower expression (homoeolog-suppressed) from a single homoeolog respecting the other two, similar to the findings in other crops like *Brassica napus* (36.5%) and bread wheat (~30%) [45, 46]. Although the genes with homoeolog expression bias were not significantly enriched in any biological process, their distributions were not stochastic, with a preference to locate in the regions near chromosome ends that showed higher recombination rates than more centric sequences in flowering plants [47]. In addition, we found that the non-balanced triads showed subtly higher Ka/Ks values than the balanced triads, suggesting that the genes with homoeolog expression bias might be under more relaxed selection pressure and have slightly higher evolutionary rates.

### Disease resistance and banana breeding

Plant pathogens continue to be a significant challenge to banana yields. Over recent decades, banana production has been severely affected by the soil-borne fungus *Foc*, which is the causal agent of the fusarium wilt of banana. The pathogen can be transmitted by plant material, water, and soil [48]. Although human societies have made many efforts to manage fusarium wilt of banana, none of these controls has been sufficiently effective for sustainable production of susceptible varieties in *Foc*-infested soils [49]. During past decades, Cavendish plantations have been threatened by tropical race 4 (*Foc*-TR4), a new strain of *Fusarium*. Recognition of chromosome regions that confer *Foc* resistance will facilitate marker-assisted selection in banana breeding schemes. R genes encode proteins that detect pathogens, thereby playing a key role in crops’ remarkable immune responses. Here we found that considerably fewer resistance genes were identified in ‘Baxijiao’ than in wild banana *M. acuminata*. Recently, Chen *et al.* [44] have identified a major quantitative trait loci (QTL)controlling resistance to the subtropical race 4 (STR4, affecting Cavendish bananas in the subtropics) of *Foc* in *M. acuminata* ssp. *malaccensis*. This QTL is located on the distal end of the long arm of chromosome 3 (36.20–42.50 Mb), corresponding to the regions including two NLR gene clusters (36.32–36.95 Mb and 40.22–40.98 Mb) found in the MAv4 genome [7]. Interestingly, we found that in the relative gene clusters in the ‘Baxijiao’ genome, nearly all NLR loci were located in the intergenic regions, indicating that they might be pseudogenized. Given this QTL-controlling resistance to STR4, the presence of much fewer NLR genes in these gene clusters could be responsible for *Foc* susceptibility of ‘Baxijiao’. This finding further confirmed the distal end of the long arm of chromosome 3 as an important region in mediating *Foc* resistance in banana. The pseudogenized NLR loci were also identified in the gene clusters in chromosome 10, indicating a possible involvement of these genomic regions in pathogen resistance too. Interestingly, the patterns of expressed NLR loci differed among the three haploid assemblies. For instance, nearly all NLR loci in a gene cluster in chromosome 3 remained functional in BXJ3, while the NLR loci in other gene clusters in the same chromosome were almost pseudogenized in BXJ1 and BXJ2; in addition, the NLR loci in the gene cluster in chromosome 10 were totally pseudogenized in BXJ2, but most loci remained functional in the other two haploid assemblies. These findings lend further support that the ‘Baxijiao’ subgenomes were of different origins, and suggested that they might contribute differently to disease resistance. The different patterns of expressed NLR loci will therefore serve as reliable references for accurate banana breeding programs in future. To summarize, the R gene clusters in chromosomes 3 and 10 might be potential targets to perform the molecular analysis of resistance in banana, which will require further research.

In conclusion, we present a high-quality T2T and haplotype-resolved reference genome of the Cavendish banana ‘Baxijiao’. The low nucleotide sequence collinearity and the differences among the three haploid assemblies closely mirror the complicated domestication history of the cultivar. Some enriched biological processes of ‘Baxijiao’-specific gene families were found to be related to fruit quality, parthenocarpy, and sterility of the cultivar. In addition, the presence of much fewer R genes in the ‘Baxijiao’ genome than in *M. acuminata* genome shed light on the possible mechanisms of fusarium wilt susceptibility of this cultivar. This genome assembly will provide a valuable reference for understanding domestication, molecular breeding and genetic improvement for banana.

## Materials and methods

### Plant materials, DNA extraction, and sequencing

Plant materials of Cavendish banana (‘Baxijiao’) were collected in South China Botanical Garden, Chinese Academy of Sciences. Tissue samples including fresh roots, leaves, flowers, and fruits were harvested, then they were immediately frozen in liquid nitrogen and preserved at −80°C for further DNA/RNA extraction. Using the CTAB method, high-quality genomic DNA was extracted from leaf tissue samples. The concentration and purity of the extracted DNA were evaluated using a Qubit fluorometer (Thermo Fisher Scientific). DNA integrity was checked by gel electrophoresis.

For PacBio HiFi sequencing, a standard SMRTbell library was constructed with SMRTbell Express Template Prep Kit 2.0 according to the manufacturer’s recommendations (Pacific Biosciences, CA, USA). The library was then sequenced on a PacBio Sequel II system. The Oxford Nanopore SQK-LSK109 kit was used to prepare the ONT library following the manufacturers’ instructions, then a Nanopore PromethION platform was used for sequencing. For Hi-C sequencing, the genomic DNA was cross-linked with formaldehyde and extracted for Hi-C library preparation. The library was paired-end sequenced on an Illumina NovaSeq platform (Illumina, CA, USA). For Illumina sequencing, paired-end 150-bp reads were generated on the Illumina NovaSeq platform. For RNAseq, total RNA was isolated from all tissue samples using the NEBNext^®^ Ultra^™^ II Directional RNA Library Prep Kit for Illumina^®^ (New England Biolabs, MA, USA). Paired-end 150-bp reads were also generated on the Illumina NovaSeq platform. All sequencing was carried out at Anhui Double Helix Gene Technology Co., Ltd (Anhui, China).

### Haplotype-resolved reference genome assembly and gap filling

Consensus reads (HiFi reads) were produced through CCS software (https://github.com/PacificBiosciences/ccs) with default parameters. Hi-C reads were filtered by fastp version 0.23.2 [50]. The obtained highly accurate HiFi reads were first used to estimate genome size and heterozygous rate with GenomeScope version 2.0 [18], based on K-mer counts produced by jellyfish [51]. Subsequently, HiFi reads and Hi-C reads were *de novo* assembled to generate the draft contig genomes of ‘Baxijiao’ using hifiasm v0.16.1 [19], which included two primary haplotypes: a first primary haploid assembly with smaller genome size and a larger one containing unphased contigs from two haploid genomes. Hi-C data were then used to anchor and remove some short contigs for the primary genome. Briefly, ragtag v2.1.0 [52] was first used to sort, orientate, and cluster the first haploid contigs guided by a T2T version of *M. acuminata* ssp. *malaccensis* genome (X. Liu *et al.*, unpublished results). Meanwhile, the contigs were anchored into 11 pseudo-chromosomes by using Juicer v1.6 [53] and 3D-DNA v180922 [54] in turn. Besides, JuiceBox v2.20.00 [21] was introduced for visualizing Hi-C data and manual modification in order to obtain the first haploid assembly containing 11 pseudo-chromosomes. Then the same workflow combining ragtag, Juicer, 3D-DNA and JuiceBox was applied to the other primary haplotype with unphased contigs, and the redundant Hi-C signals were used to separate the second and the third haploid assemblies. The tools agp2assembly.py and 01_2assemblyto2.py in JuiceBox were then used to group the contigs from the three haploid assemblies together and arrange them in a sequential manner. Meanwhile, through examination on the final turn, the file containing the combined contigs and assemblies from all three haploid assemblies was introduced to JuiceBox, and some excess contigs belonging to the third haploid assembly could be manually adjusted according to Hi-C signals. Finally, we generated 33 pseudo-chromosomes based on our high-covered Hi-C data and high-accurate HiFi reads. For gap filling, the ONT ultra-long reads were first used for *de novo* assembly by NextDenovo (https://github.com/Nextomics/NextDenovo), and polished by Nextpolish [55] based on the HiFi reads and the Illumina reads with default parameters. Then the ONT polished contigs were mapped to the primary genome using minimap2 v2.24-r1122 [56]. Because there are only 144 gaps in the primary genome, we examined the breakpoint locations with the Integrative Genomics Viewer (IGV) [57] tool and manually filled the gaps according to the alignments. After filling gaps, a nearly complete and phased reference genome named BXJ was generated. The scheme showing ‘Baxijiao’ genome assembly is presented in Fig. S2 (see online supplementary material). Telomere regions were identified using TIDK v0.2.1 (https://github.com/tolkit/telomeric-identifier). Centromeric regions were identified based on the results of Tandem Repeats Finder v4.09 [20] using default parameters and TE libraries.

### Assembly validation and quality assessment

HiFi and Illumina reads were used to evaluate the accuracy of BXJ. HiFi reads were mapped to the assembly using minimap2, while Illumina reads were aligned with BWA v0.7.17-r1188 [58]. The mapping rate was calculated using BamTools v2.5.1 [59]. The LTR Assembly Index (LAI) calculated from LTR_retriever v2.9.0 [60] was introduced to assess the genome assembly quality. BUSCO v5.4.3 [22] was used for the evaluation of assembly completeness of the haplotype-resolved genome with the ‘embryophyta_odb 10’ database.

### Whole genome alignment and syntenic analysis

The syntenic relationships between haploid assemblies were determined using nucmer v4.0.0rc1 [61] with default parameters. Delta-filter was then launched with parameters ‘-i 90 -l 15000’. LINKVIEW v1.1 (https://github.com/YangJianshun/LINKVIEW) was used for visualizing syntenic relationships at chromosome level. In order to further check large inversion regions, we used IGV and JuiceBox to examine the breakpoint regions of inversions according to the validation methods mentioned in Tang *et al.* [62] and Zhou *et al.* [63] (Figs S8–S9, see online supplementary material). MCscan [23] pipeline was used to find structure variations among the BXJ haploid assemblies. Briefly, the ‘jcvi.compara.catalog’ module with parameter ‘—cscore = 0.99’ was performed to find ortholog; then, ‘jcvi.compara.synteny’ module with ‘—minspan = 30’ was used to build the syntenic regions; finally, ‘jcvi.graphics.karyotype’ module was carried out to visualize the syntenic relationships.

### Genome repeats and gene annotation

TEs libraries were identified by RepeatModeler v2.0.1 [64] with default parameters. RepeatMasker v4.1.1 (http://repeatmasker.org/) was used to identify repeats with the consensus TEs libraries. Then the repeat-masked genome was imported to MAKER3 v3.01.03 [65] for gene annotation. All high-confidence Swiss-prot protein sequences were imported for homology prediction by Protein2Genome with parameters protein2genome = 1. Transcripts from the four tissues were used by Est2Genome with parameters est2genome = 1, then the ab-initio prediction model was constructed by AUGUSTUSv3.3.2. AUGUSTUS models were tested based on two divided datasets, one for test and another for evaluation, and results showed our models were sufficiently reliable. Finally, the MAKER3 pipeline was run again to obtain high-quality gene annotations combining homology predictions, transcripts evidence predictions, and *de novo* predictions. Funannotate v1.8.7 (https://github.com/nextgenusfs/funannotate) was used to functionally annotate genes based on blast results from these databases: Pfam, UniProt, GO, KEGG, InterPro, dbCAN, EggNOG, and MEROPS.

### Gene family expansion and contraction

Genes families in ‘Baxijiao’ and 15 other species in monocots were characterized using Orthofinder v2.5.4 [66] by comparing their protein-coding gene sequences. The single-copy ortholog sequences, which strictly existed in all above species, were then aligned using mafft v7.310 [67]. Subsequently, the concatenated sequences were subjected to phylogenetic analysis using RAxML v8.2.12 [68] with 2000 bootstraps. Once the phylogenetic tree was constructed, MCMCTree [69] was applied to estimate divergence times and a total of nine species pairs were selected as calibration points, whose estimated divergence times were acquired from the TimeTree website (Table S17, see online supplementary material). Two MCMCTree runs were performed to ensure the convergence of the divergence time estimation. CAFE v5.0 [70] was carried out to identify potential expanded and contracted gene families. For significantly expanded and contracted gene sets, GO enrichment analysis was conducted using TBtools v1.108 [71].

### Identification of resistance genes

Heritable genetic variation for disease resistance in crop species is often affected by disease resistance (R) genes. RGAugury v2.2 [25] was used to predict resistance gene analogs (RGAs) in ‘Baxijiao’ and *M. acuminata*. The search for NLR loci was carried out by NLR-Annotator [26], a tool for *de novo* genome annotation of NLR loci independent of transcript support. A potential NLR locus might correspond to a complete or partial gene and might also be the trace of a pseudogenized sequence.

### Homoeolog expression patterns in ‘Baxijiao’

After filtering by fastp, RNAseq reads were aligned to the haplotype-resolved BXJ genome using HISAT2 v2.2.1 [72]. featureCounts v2.0.4 [73] was then used to quantify RNAseq data as counts. To identify alleles in the BXJ genome, we applied DIAMOND v2.0.15 [74] to align the protein sequences among ‘Baxijiao’ haploid assemblies and MAv4, and obtain three types of alleles: three alleles paired with each other (1:1:1 correspondence), two alleles paired with each other, and the remaining, based on the identity scores. To explore patterns of homoeolog expression, we focused on the gene triads that had a 1:1:1 correspondence across the three haploid assemblies. A homoeolog with expression level >0.5 transcripts per million (TPM) in at least one tissue was considered expressed. Relative expression levels of the BXJ1, BXJ2, and BXJ3 homoeologs across triads were standardized following Ramírez-González *et al.* [46], based on the normalized absolute TPM and the relative expression level of each homoeolog was normalized as expression*_BXJ1_* = TPM(*BXJ1*) / (TPM(*BXJ1*) + TPM(*BXJ2*) + TPM(*BXJ3*)), expression*_BXJ2_* = TPM(*BXJ2*) / (TPM(*BXJ1*) + TPM(*BXJ2*) + TPM(*BXJ3*)), and expression*_BXJ3_* = TPM(*BXJ3*) / (TPM(*BXJ1*) + TPM(*BXJ2*) + TPM(*BXJ3*)). The relative expression level of each homoeolog per triad was introduced to plot the ternary diagrams using the R package *ggtern* [75]. Based on these plots, we described seven groups of homoeolog expression bias: a balanced group, with similar relative expression levels from the three homoeologs, and six homoeolog-dominant or homoeolog-suppressed groups, defined on the basis of the higher or lower relative expression levels from a single homoeolog compared to the other two (Table S18, see online supplementary material). KAKS Calculator v2.0 [76] was used to calculate Ka/Ks values of different expression bias categories. For homoeolog-dominant or homoeolog-suppressed genes, GO enrichment analyses were carried out using TBtools.

## Supplementary Material

Web_Material_uhad153

## Data Availability

The Cavendish (BXJ) raw genome sequencing reads are available from the National Center for Biotechnology Information (NCBI) under project ID PRJNA957115 and the National Genomics Data Center (NGDC) under project ID PRJCA016940. The Cavendish assembly and annotation files have been submitted to FigShare [77].

## References

[1] Perrier X , De Langhe E, Donohue M et al. Multidisciplinary perspectives on banana (*Musa* spp.) domestication. Proc Natl Acad Sci U S A. 2011;108:11311–8 21730145 10.1073/pnas.1102001108 PMC3136277

[2] Davey MW , Gudimella R, Harikrishna JA et al. A draft *Musa balbisiana* genome sequence for molecular genetics in polyploid, inter- and intra-specific *Musa* hybrids. BMC Genomics. 2013;14:683 24094114 10.1186/1471-2164-14-683 PMC3852598

[3] FAO . World Food and Agriculture-Statistical Yearbook 2020. (FAO, 2020)

[4] Dita M , Barquero M, Heck D et al. Fusarium wilt of banana: current knowledge on epidemiology and research needs toward sustainable disease management. Front Plant Sci. 2018;9:1468 30405651 10.3389/fpls.2018.01468 PMC6202804

[5] D'Hont A , Denoeud F, Aury JM et al. The banana (*Musa acuminata*) genome and the evolution of monocotyledonous plants. Nature. 2012;488:213–7 22801500 10.1038/nature11241

[6] Martin G , Baurens FC, Droc G et al. Improvement of the banana “*Musa acuminata*” reference sequence using NGS data and semi-automated bioinformatics methods. BMC Genomics. 2016;17:243 26984673 10.1186/s12864-016-2579-4 PMC4793746

[7] Belser C , Baurens FC, Noel B et al. Telomere-to-telomere gapless chromosomes of banana using nanopore sequencing. Commun Biol. 2021;4:1047 34493830 10.1038/s42003-021-02559-3 PMC8423783

[8] Wang Z , Miao H, Liu J et al. *Musa balbisiana* genome reveals subgenome evolution and functional divergence. Nat Plants. 2019;5:810–21 31308504 10.1038/s41477-019-0452-6 PMC6784884

[9] Wu W , Yang YL, He WM et al. Whole genome sequencing of a banana wild relative *Musa itinerans* provides insights into lineage-specific diversification of the *Musa* genus. Sci Rep. 2016;6:31586 27531320 10.1038/srep31586 PMC4987669

[10] Belser C , Istace B, Denis E et al. Chromosome-scale assemblies of plant genomes using nanopore long reads and optical maps. Nat Plants. 2018;4:879–87 30390080 10.1038/s41477-018-0289-4

[11] Li Z , Wang J, Fu Y et al. The *Musa troglodytarum* L. genome provides insights into the mechanism of non-climacteric behaviour and enrichment of carotenoids. BMC Biol. 2022;20:186 36002843 10.1186/s12915-022-01391-3 PMC9400310

[12] Wang ZF , Rouard M, Droc G et al. Genome assembly of *Musa beccarii* shows extensive chromosomal rearrangements and genome expansion during evolution of Musaceae genomes. GigaScience. 2022;12:giad005 36807539 10.1093/gigascience/giad005 PMC9941839

[13] Shi X , Cao S, Wang X et al. The complete reference genome for grapevine (*Vitis vinifera* L.) genetics and breeding. Hortic Res. 2023;10:uhad061 37213686 10.1093/hr/uhad061 PMC10199708

[14] Cenci A , Sardos J, Hueber Y et al. Unravelling the complex story of intergenomic recombination in ABB allotriploid bananas. Ann Bot. 2021;127:7–20 32104882 10.1093/aob/mcaa032 PMC7750727

[15] Martin G , Cardi C, Sarah G et al. Genome ancestry mosaics reveal multiple and cryptic contributors to cultivated banana. Plant J. 2020;102:1008–25 31930580 10.1111/tpj.14683 PMC7317953

[16] Martin G , Cottin A, Baurens FC et al. Interspecific introgression patterns reveal the origins of worldwide cultivated bananas in New Guinea. Plant J. 2023;113:802–18 36575919 10.1111/tpj.16086

[17] Drenth A , Kema G. The vulnerability of bananas to globally emerging disease threats. Phytopathology. 2021;111:2146–61 34231377 10.1094/PHYTO-07-20-0311-RVW

[18] Ranallo-Benavidez TR , Jaron KS, Schatz MC. GenomeScope 2.0 and Smudgeplot for reference-free profiling of polyploid genomes. Nat Commun. 2020;11:1432 32188846 10.1038/s41467-020-14998-3 PMC7080791

[19] Cheng H , Concepcion GT, Feng X et al. Haplotype-resolved *de novo* assembly using phased assembly graphs with hifiasm. Nat Methods. 2021;18:170–5 33526886 10.1038/s41592-020-01056-5 PMC7961889

[20] Benson G . Tandem repeats finder: a program to analyze DNA sequences. Nucleic Acids Res. 1999;27:573–80 9862982 10.1093/nar/27.2.573 PMC148217

[21] Durand NC , Robinson JT, Shamim MS et al. Juicebox provides a visualization system for hi-C contact maps with unlimited zoom. Cell Syst. 2016;3:99–101 27467250 10.1016/j.cels.2015.07.012 PMC5596920

[22] Manni M , Berkeley MR, Seppey M et al. BUSCO update: novel and streamlined workflows along with broader and deeper phylogenetic coverage for scoring of eukaryotic, prokaryotic, and viral genomes. Mol Biol Evol. 2021;38:4647–54 34320186 10.1093/molbev/msab199 PMC8476166

[23] Tang H , Bowers JE, Wang X et al. Synteny and collinearity in plant genomes. Science. 2008;320:486–8 18436778 10.1126/science.1153917

[24] Wang J , Song W, Chai J. Structure, biochemical function, and signaling mechanism of plant NLRs. Mol Plant. 2023;16:75–95 36415130 10.1016/j.molp.2022.11.011

[25] Li P , Quan X, Jia G et al. RGAugury: a pipeline for genome-wide prediction of resistance gene analogs (RGAs) in plants. BMC Genomics. 2016;17:852 27806688 10.1186/s12864-016-3197-x PMC5093994

[26] Steuernagel B , Witek K, Krattinger SG et al. The NLR-annotator tool enables annotation of the intracellular immune receptor repertoire. Plant Physiol. 2020;183:468–82 32184345 10.1104/pp.19.01273 PMC7271791

[27] Perrier X , Bakry F, Carreel F et al. Combining biological approaches to shed light on the evolution of edible bananas. Ethnobot Res Appl. 2009;7:199–216

[28] Martin G , Baurens FC, Hervouet C et al. Chromosome reciprocal translocations have accompanied subspecies evolution in bananas. Plant J. 2020;104:1698–711 33067829 10.1111/tpj.15031 PMC7839431

[29] Dupouy M , Baurens FC, Derouault P et al. Two large reciprocal translocations characterized in the disease resistance-rich *burmannica* genetic group of *Musa acuminata*. Ann Bot. 2019;124:319–29 31241133 10.1093/aob/mcz078 PMC6758587

[30] Sardos J , Breton C, Perrier X et al. Hybridization, missing wild ancestors and the domestication of cultivated diploid bananas. Front Plant Sci. 2022;13:969220 36275535 10.3389/fpls.2022.969220 PMC9586208

[31] Hippolyte I , Jenny C, Gardes L et al. Foundation characteristics of edible *Musa* triploids revealed from allelic distribution of SSR markers. Ann Bot. 2012;109:937–51 22323428 10.1093/aob/mcs010 PMC3310492

[32] Cirilli M , Bassi D, Ciacciulli A. Sugars in peach fruit: a breeding perspective. Hortic Res. 2016;3:15067 26816618 10.1038/hortres.2015.67 PMC4720000

[33] Fan Z , Hasing T, Johnson TS et al. Strawberry sweetness and consumer preference are enhanced by specific volatile compounds. Hortic Res. 2021;8:66 33790262 10.1038/s41438-021-00502-5 PMC8012349

[34] Soares CA , Peroni-Okita FH, Cardoso MB et al. Plantain and banana starches: granule structural characteristics explain the differences in their starch degradation patterns. J Agric Food Chem. 2011;59:6672–81 21591784 10.1021/jf201590h

[35] Cordenunsi-Lysenko BR , Nascimento JRO, Castro-Alves VC et al. The starch is (not) just another brick in the wall: the primary metabolism of sugars during banana ripening. Front Plant Sci. 2019;10:391 31001305 10.3389/fpls.2019.00391 PMC6454214

[36] Liu TT , Yang TS. Optimization of solid-phase microextraction analysis for studying change of headspace flavor compounds of banana during ripening. J Agric Food Chem. 2002;50:653–7 11829624 10.1021/jf010891+

[37] Wyllie SG , Fellman JK. Formation of volatile branched chain esters in bananas (*Musa sapientum* L.). J Agric Food Chem. 2000;48:3493–6 10956138 10.1021/jf0001841

[38] de Vasconcelos Facundo HV , dos Santos D, dos Santos Dias CT et al. Influence of different banana cultivars on volatile compounds during ripening in cold storage. Food Res Int. 2012;49:626–33

[39] Zhu X , Li Q, Li J et al. Comparative study of volatile compounds in the fruit of two banana cultivars at different ripening stages. Molecules. 2018;23:2456 30257494 10.3390/molecules23102456 PMC6222428

[40] Denham T , Barton H, Castillo C et al. The domestication syndrome in vegetatively propagated field crops. Ann Bot. 2020;125:581–97 31903489 10.1093/aob/mcz212 PMC7102979

[41] Pacini E , Franchi G, Hesse M. The tapetum: its form, function, and possible phylogeny in Embryophyta. Plant Syst Evol. 1985;149:155–85

[42] Tariq N , Yaseen M, Xu D et al. Rice anther tapetum: a vital reproductive cell layer for sporopollenin biosynthesis and pollen exine patterning. Plant Biol. 2023;25:233–45 36350096 10.1111/plb.13485

[43] Li W , Dita M, Wu W et al. Resistance sources to *Fusarium oxysporum* f. sp. *cubense* tropical race 4 in banana wild relatives. Plant Pathol. 2015;64:1061–7

[44] Chen A , Sun J, Martin G et al. Identification of a major QTL-controlling resistance to the subtropical race 4 of *Fusarium oxysporum* f. sp. *cubense* in *Musa acuminata* ssp. *malaccensis*. Pathogens. 2023;12:289 36839561 10.3390/pathogens12020289 PMC9964652

[45] Wu J , Lin L, Xu M et al. Homoeolog expression bias and expression level dominance in resynthesized allopolyploid *Brassica napus*. BMC Genomics. 2018;19:586 30081834 10.1186/s12864-018-4966-5 PMC6080508

[46] Ramírez-González RH , Borrill P, Lang D et al. The transcriptional landscape of polyploid wheat. Science. 2018;361:eaar6089 30115782 10.1126/science.aar6089

[47] Brazier T , Glemin S. Diversity and determinants of recombination landscapes in flowering plants. PLoS Genet. 2022;18:e1010141 36040927 10.1371/journal.pgen.1010141 PMC9467342

[48] Siamak SB , Zheng S. Banana Fusarium wilt (*Fusarium oxysporum* f. sp. *cubense*) control and resistance, in the context of developing wilt-resistant bananas within sustainable production systems. Hortic Plant J. 2018;4:208–18

[49] Ploetz RC . Panama disease: an old nemesis rears its ugly: head part 2. The cavendish era and beyond. Plant Health Progress. 2006;7:36

[50] Chen S , Zhou Y, Chen Y et al. Fastp: an ultra-fast all-in-one FASTQ preprocessor. Bioinformatics. 2018;34:i884–90 30423086 10.1093/bioinformatics/bty560 PMC6129281

[51] Marcais G , Kingsford C. A fast, lock-free approach for efficient parallel counting of occurrences of k-mers. Bioinformatics. 2011;27:764–70 21217122 10.1093/bioinformatics/btr011 PMC3051319

[52] Alonge M , Soyk S, Ramakrishnan S et al. RaGOO: fast and accurate reference-guided scaffolding of draft genomes. Genome Biol. 2019;20:224 31661016 10.1186/s13059-019-1829-6 PMC6816165

[53] Durand NC , Shamim MS, Machol I et al. Juicer provides a one-click system for analyzing loop-resolution hi-C experiments. Cell Syst. 2016;3:95–8 27467249 10.1016/j.cels.2016.07.002 PMC5846465

[54] Dudchenko O , Batra SS, Omer AD et al. *De novo* assembly of the *Aedes aegypti* genome using hi-C yields chromosome-length scaffolds. Science. 2017;356:92–5 28336562 10.1126/science.aal3327 PMC5635820

[55] Hu J , Fan J, Sun Z et al. NextPolish: a fast and efficient genome polishing tool for long-read assembly. Bioinformatics. 2020;36:2253–5 31778144 10.1093/bioinformatics/btz891

[56] Li H . Minimap2: pairwise alignment for nucleotide sequences. Bioinformatics. 2018;34:3094–100 29750242 10.1093/bioinformatics/bty191 PMC6137996

[57] Thorvaldsdottir H , Robinson JT, Mesirov JP. Integrative Genomics Viewer (IGV): high-performance genomics data visualization and exploration. Brief Bioinform. 2013;14:178–92 22517427 10.1093/bib/bbs017 PMC3603213

[58] Li H , Durbin R. Fast and accurate short read alignment with burrows-wheeler transform. Bioinformatics. 2009;25:1754–60 19451168 10.1093/bioinformatics/btp324 PMC2705234

[59] Barnett DW , Garrison EK, Quinlan AR et al. BamTools: a C++ API and toolkit for analyzing and managing BAM files. Bioinformatics. 2011;27:1691–2 21493652 10.1093/bioinformatics/btr174 PMC3106182

[60] Ou S , Jiang N. LTR_retriever: a highly accurate and sensitive program for identification of long terminal repeat retrotransposons. Plant Physiol. 2018;176:1410–22 29233850 10.1104/pp.17.01310 PMC5813529

[61] Marcais G , Delcher AL, Phillippy AM et al. MUMmer4: a fast and versatile genome alignment system. PLoS Comput Biol. 2018;14:e1005944 29373581 10.1371/journal.pcbi.1005944 PMC5802927

[62] Tang D , Jia Y, Zhang J et al. Genome evolution and diversity of wild and cultivated potatoes. Nature. 2022;606:535–41 35676481 10.1038/s41586-022-04822-x PMC9200641

[63] Zhou Y , Minio A, Massonnet M et al. The population genetics of structural variants in grapevine domestication. Nat Plants. 2019;5:965–79 31506640 10.1038/s41477-019-0507-8

[64] Flynn JM , Hubley R, Goubert C et al. RepeatModeler2 for automated genomic discovery of transposable element families. Proc Natl Acad Sci U S A. 2020;117:9451–7 32300014 10.1073/pnas.1921046117 PMC7196820

[65] Campbell MS , Holt C, Moore B et al. Genome annotation and curation using MAKER and MAKER-P. Curr Protoc Bioinformatics. 2014;48:4.11.1–39 10.1002/0471250953.bi0411s48 PMC4286374 25501943

[66] Emms DM , Kelly S. OrthoFinder: phylogenetic orthology inference for comparative genomics. Genome Biol. 2019;20:238 31727128 10.1186/s13059-019-1832-y PMC6857279

[67] Katoh K , Standley DM. MAFFT multiple sequence alignment software version 7: improvements in performance and usability. Mol Biol Evol. 2013;30:772–80 23329690 10.1093/molbev/mst010 PMC3603318

[68] Stamatakis A . RAxML version 8: a tool for phylogenetic analysis and post-analysis of large phylogenies. Bioinformatics. 2014;30:1312–3 24451623 10.1093/bioinformatics/btu033 PMC3998144

[69] Dos Reis M , Zhu T, Yang Z. The impact of the rate prior on Bayesian estimation of divergence times with multiple loci. Syst Biol. 2014;63:555–65 24658316 10.1093/sysbio/syu020 PMC4055871

[70] Mendes FK , Vanderpool D, Fulton B et al. CAFE 5 models variation in evolutionary rates among gene families. Bioinformatics. 2021;36:5516–8 33325502 10.1093/bioinformatics/btaa1022

[71] Chen C , Chen H, Zhang Y et al. TBtools: an integrative toolkit developed for interactive analyses of big biological data. Mol Plant. 2020;13:1194–202 32585190 10.1016/j.molp.2020.06.009

[72] Kim D , Paggi JM, Park C et al. Graph-based genome alignment and genotyping with HISAT2 and HISAT-genotype. Nat Biotechnol. 2019;37:907–15 31375807 10.1038/s41587-019-0201-4 PMC7605509

[73] Liao Y , Smyth GK, Shi W. featureCounts: an efficient general purpose program for assigning sequence reads to genomic features. Bioinformatics. 2014;30:923–30 24227677 10.1093/bioinformatics/btt656

[74] Buchfink B , Reuter K, Drost HG. Sensitive protein alignments at tree-of-life scale using DIAMOND. Nat Methods. 2021;18:366–8 33828273 10.1038/s41592-021-01101-x PMC8026399

[75] Hamilton NE , Ferry M. Ggtern: ternary diagrams using ggplot2. J Stat Softw. 2018;87:1–17

[76] Wang D , Zhang Y, Zhang Z et al. KaKs_Calculator 2.0: a toolkit incorporating gamma-series methods and sliding window strategies. Genom Proteom Bioinform. 2010;8:77–80 10.1016/S1672-0229(10)60008-3 PMC5054116 20451164

[77] Huang HR , Liu X, Arshad R et al. Telomere-to-telomere haplotype-resolved reference genome reveals subgenome divergence and disease resistance in triploid Cavendish banana. Figshare. 2023. 10.1093/hr/uhad153 PMC10493638 37701454

