# Supplementary material for: Telomere-to-telomere haplotype-resolved reference genome reveals subgenome divergence and disease resistance in triploid Cavendish banana
Source: Hortic Res. 2023 Aug 1;10(9):uhad153. doi: 10.1093/hr/uhad153 (PMC10493638; doi:10.1093/hr/uhad153)
Supplement: Web_Material_uhad153 [file web_material_uhad153.zip › Supplementary Fig. S1-S9, Table S1-S5, S9, S17,S18.pdf]

Supplementary information for:

Telomere-to-telomere haplotype-resolved reference genome reveals subgenome divergence and disease resistance in triploid Cavendish banana

Hui-Run Huang, Xin Liu, Rida Arshad, Xu Wang, Wei-Ming Li, Yongfeng Zhou, Xue-Jun Ge

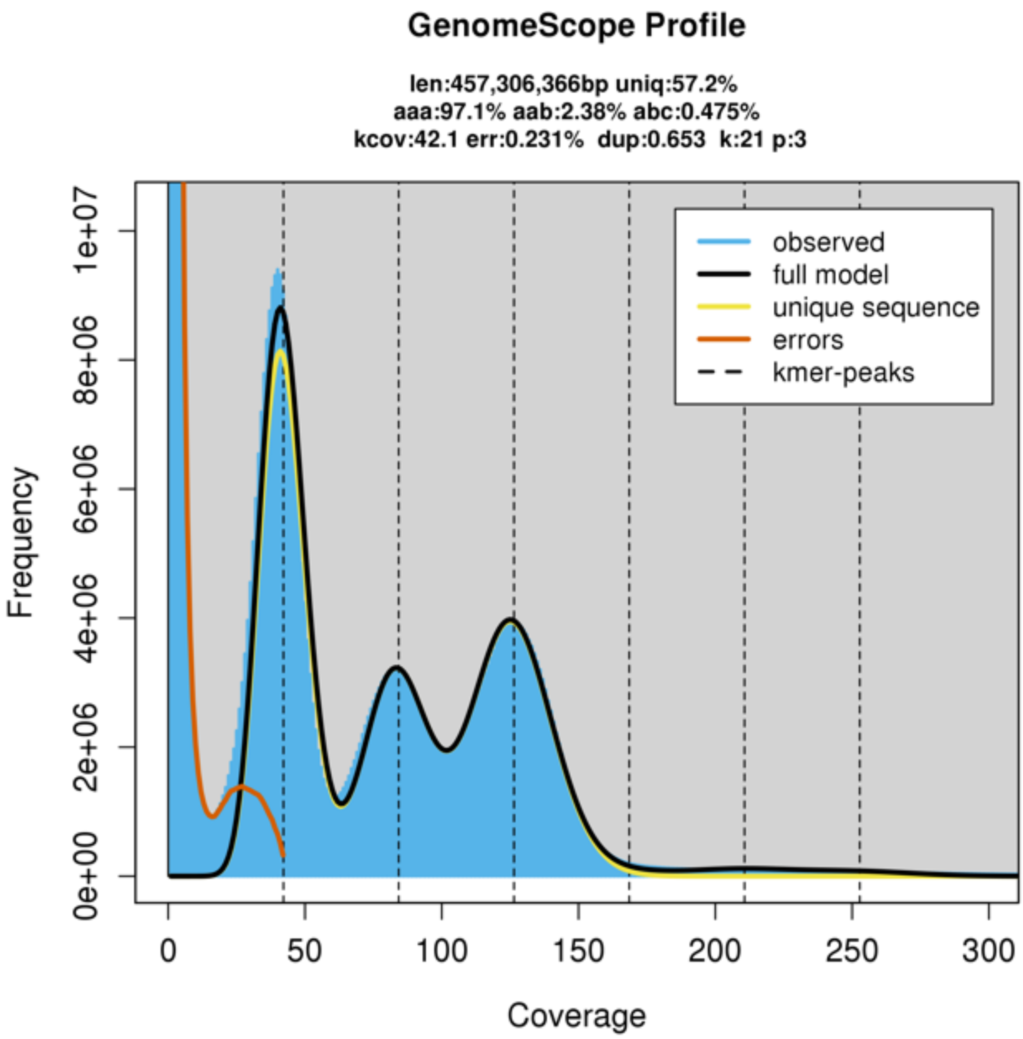

Figure S1. Estimation of genome size based on 21 *K*-mer distribution.

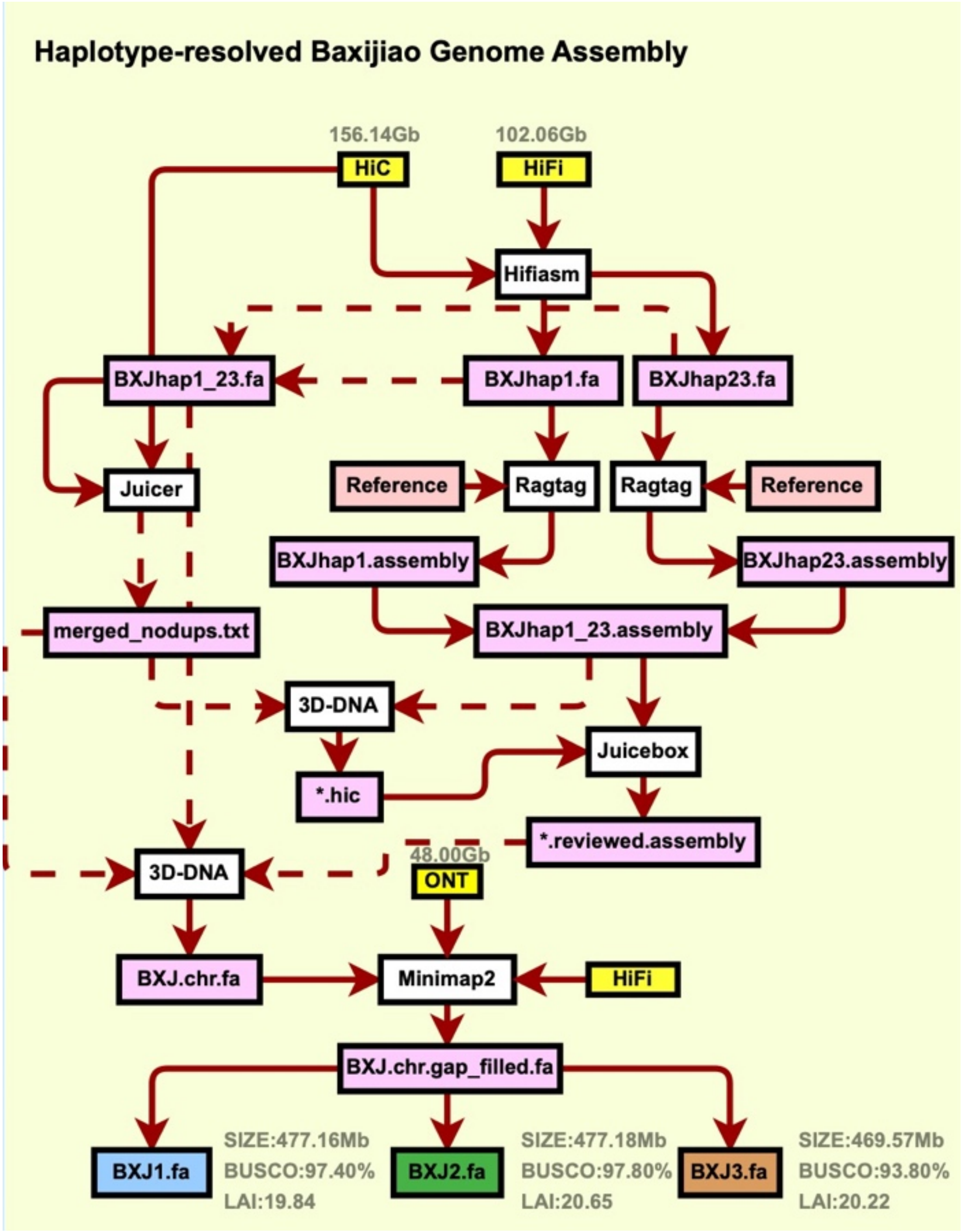

Figure S2. Pipeline of haplotype-resolved ‘Baxijiao’ genome assembly.

BXJ1

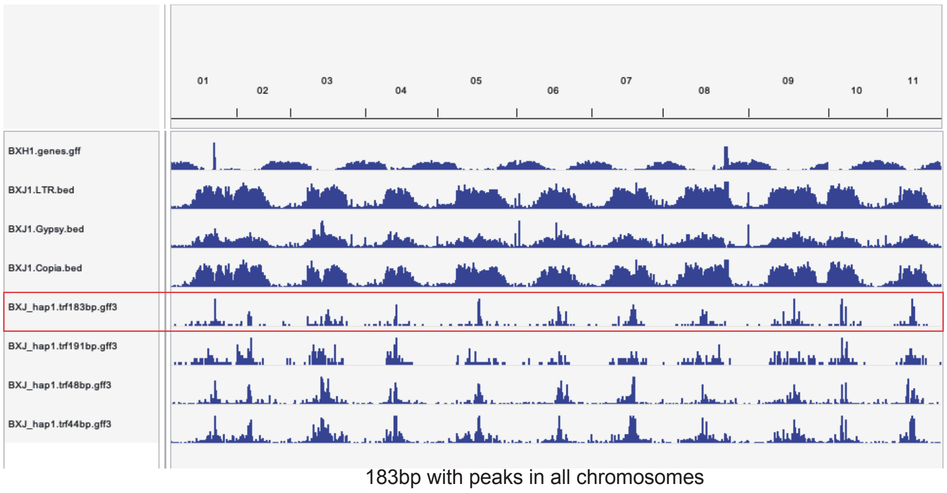

183bp with peaks in all chromosomes

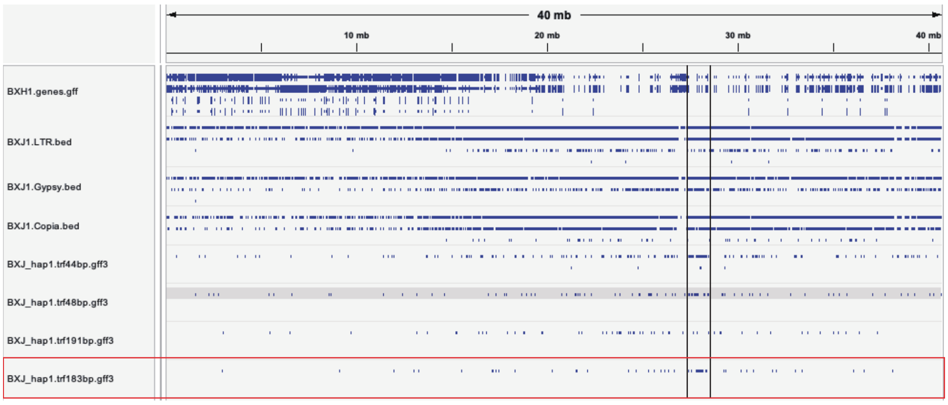

chr01

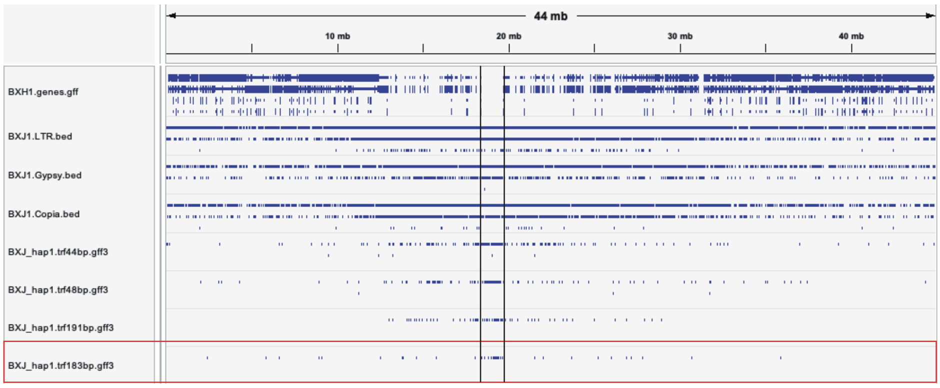

chr04

BXJ2

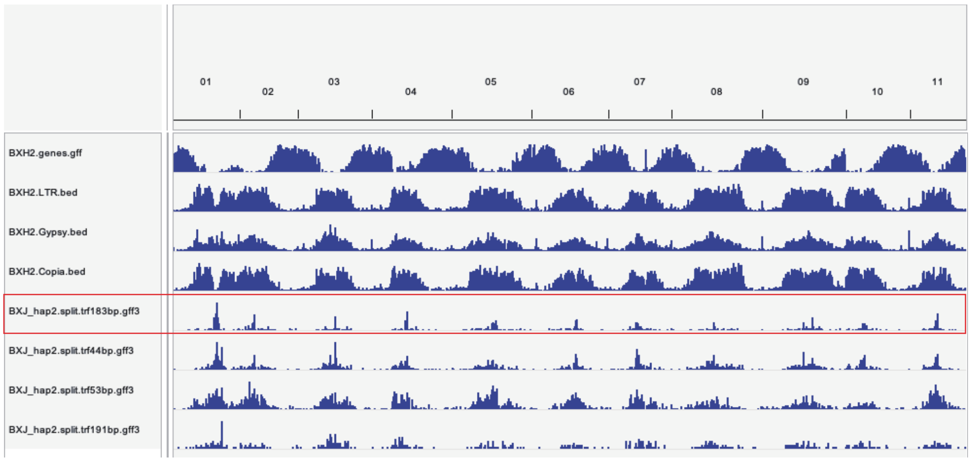

183bp with peaks in all chromosomes

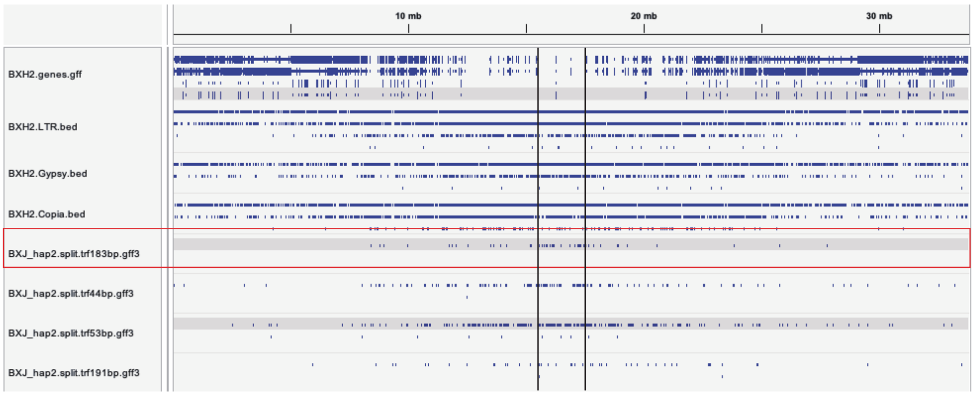

chr11

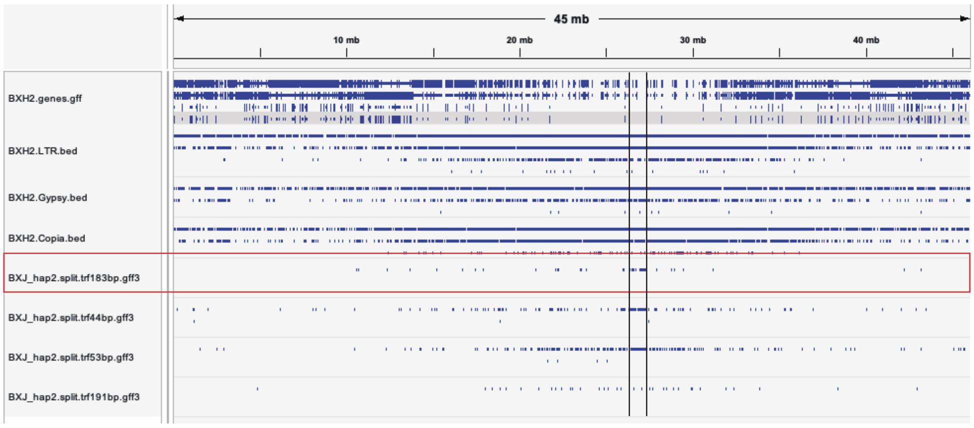

chr06

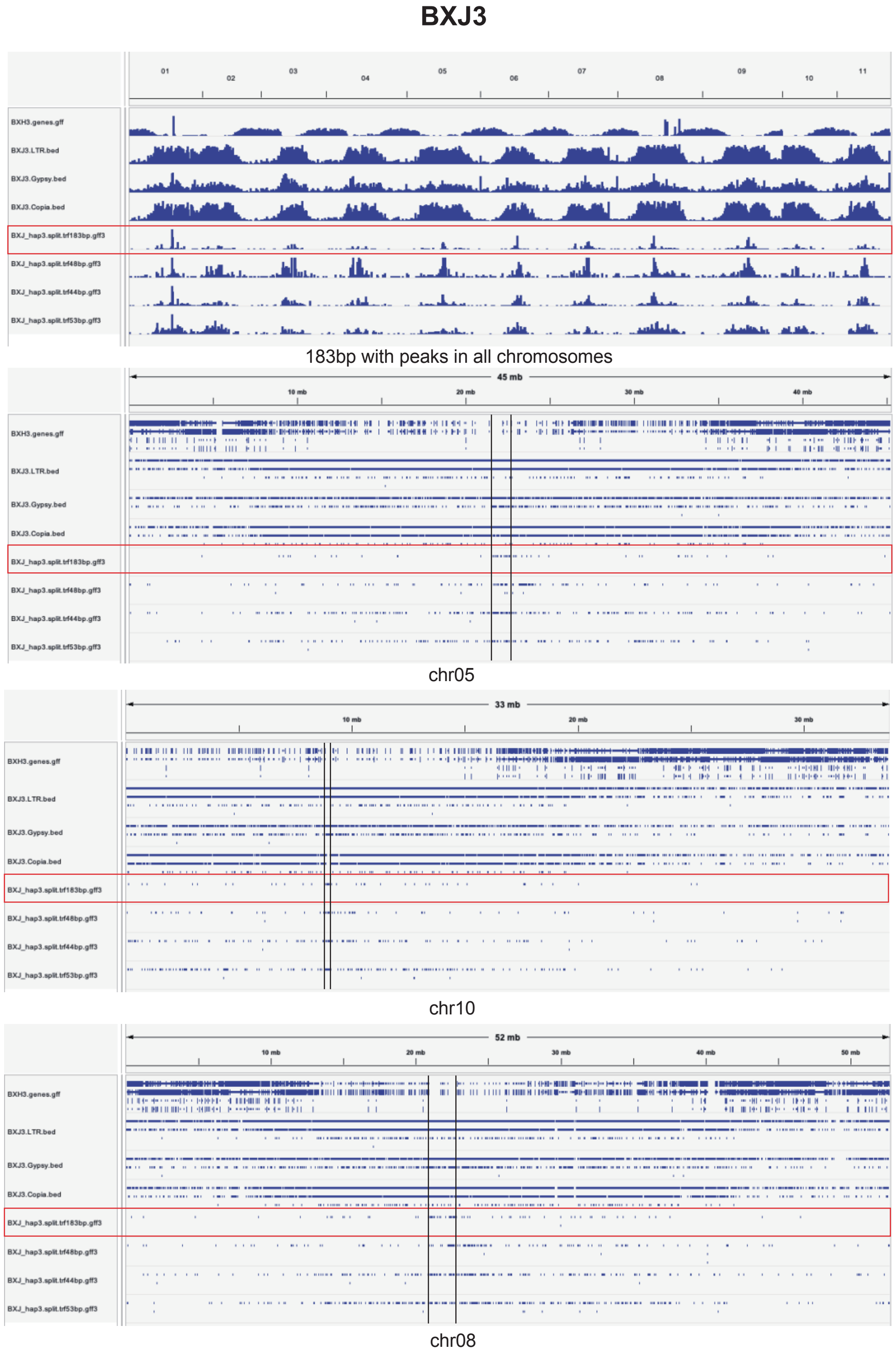

Figure S3. Potential centromere regions of the BXJ genome. 183bp TRF was considered the most possible centromeric tendem repeats units, which existed in almost all chromosomes.

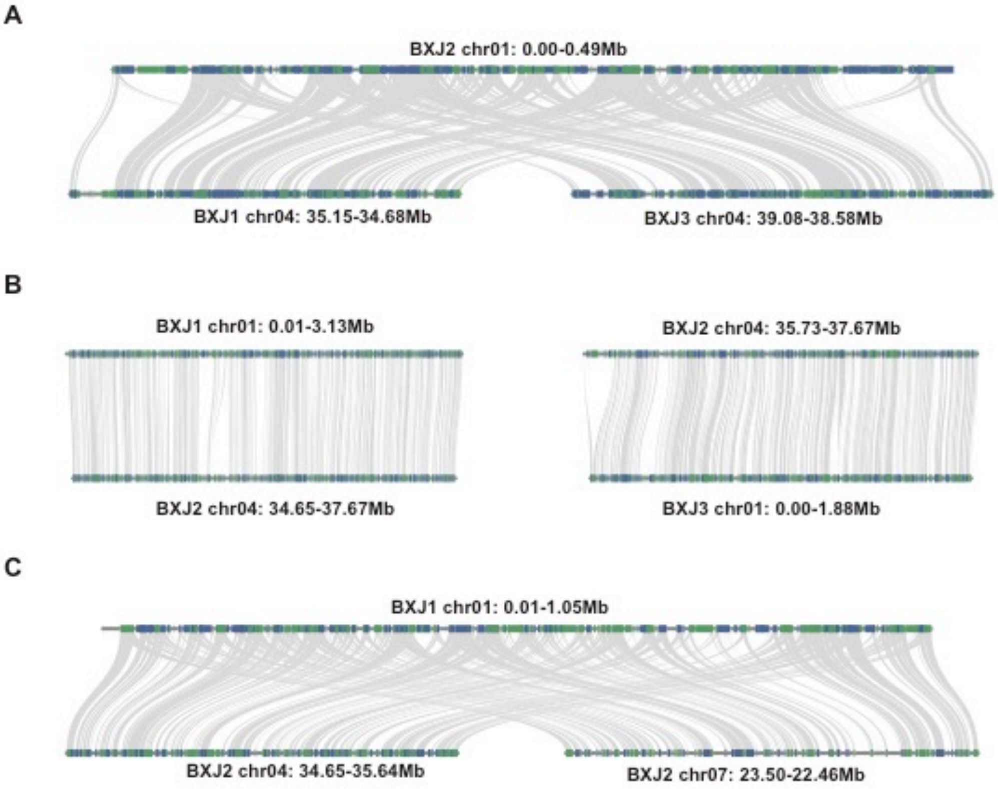

Figure S4. The large reciprocal translocations characterized among Chr01, Chr04 and Chr07.

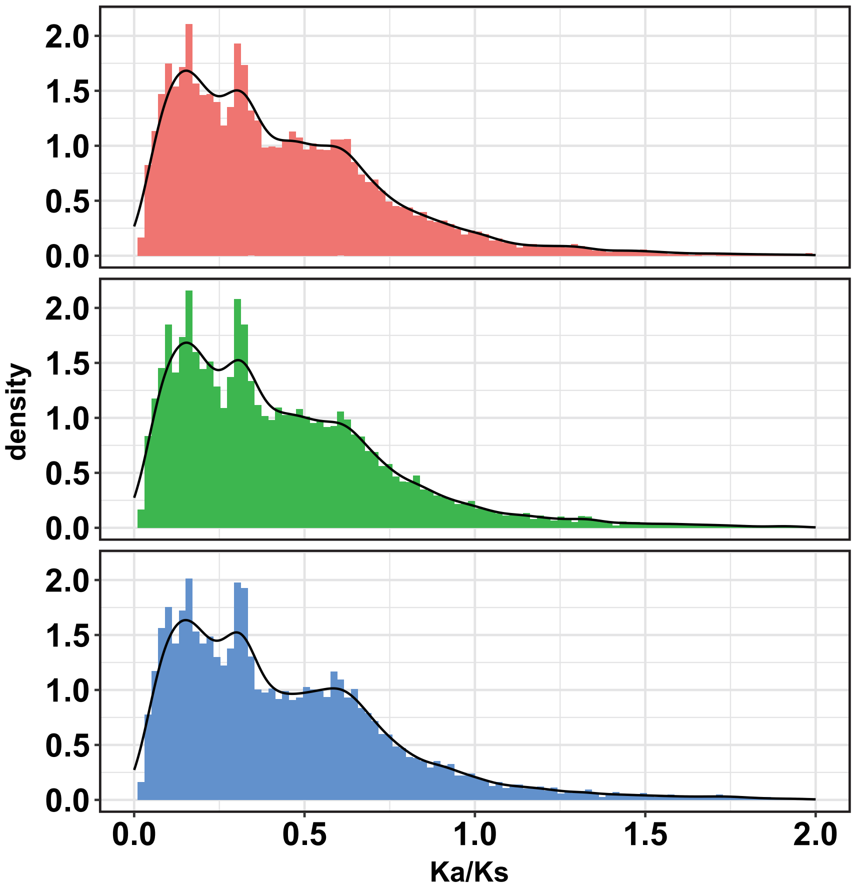

Figure S5. The distribution of Ka/Ks values of alleles between BXJ haploid assemblies and MAv4.

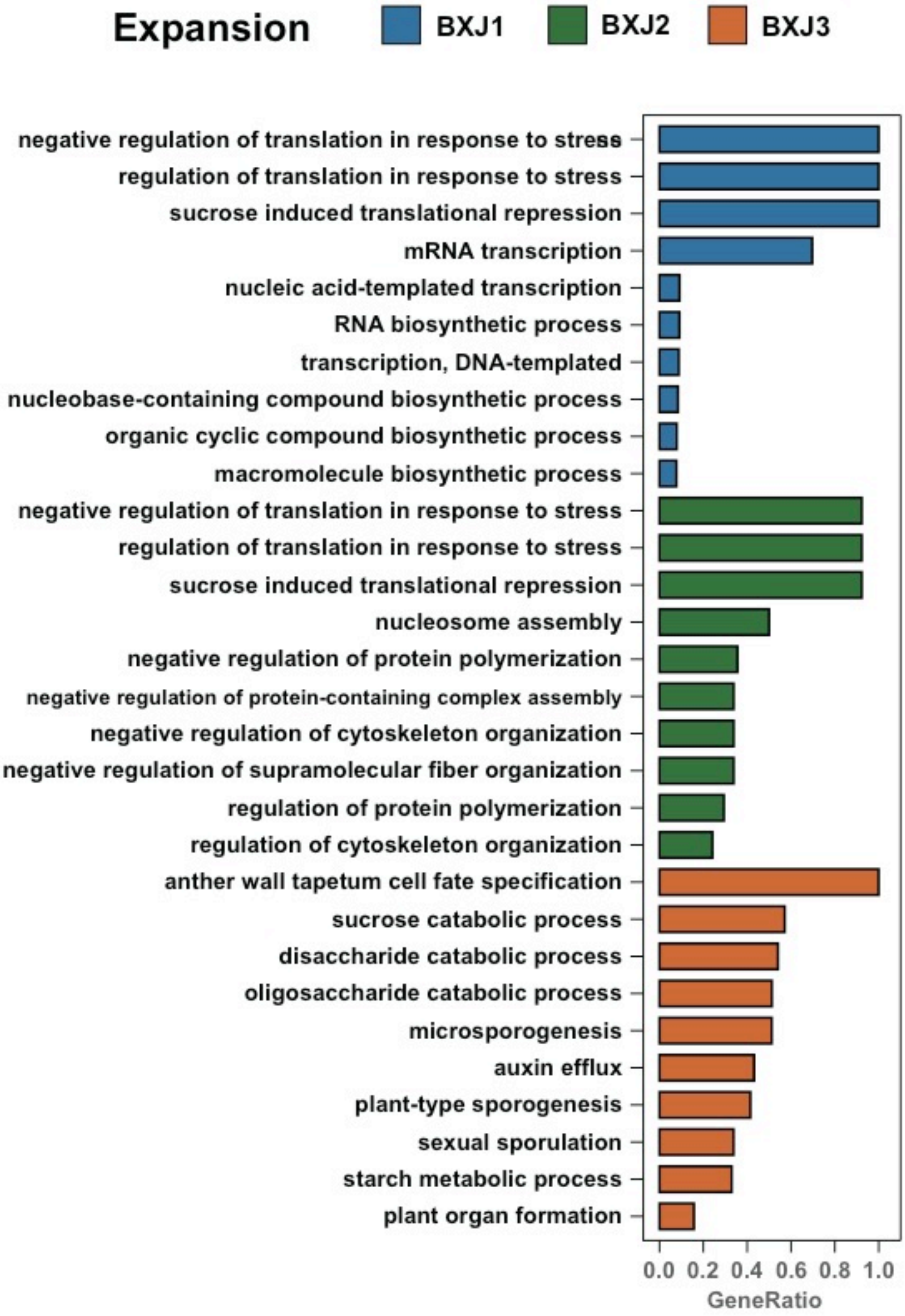

Figure S6. The top enriched biological processes for expanded genes in each haploid assembly of 'Baxijiao'.

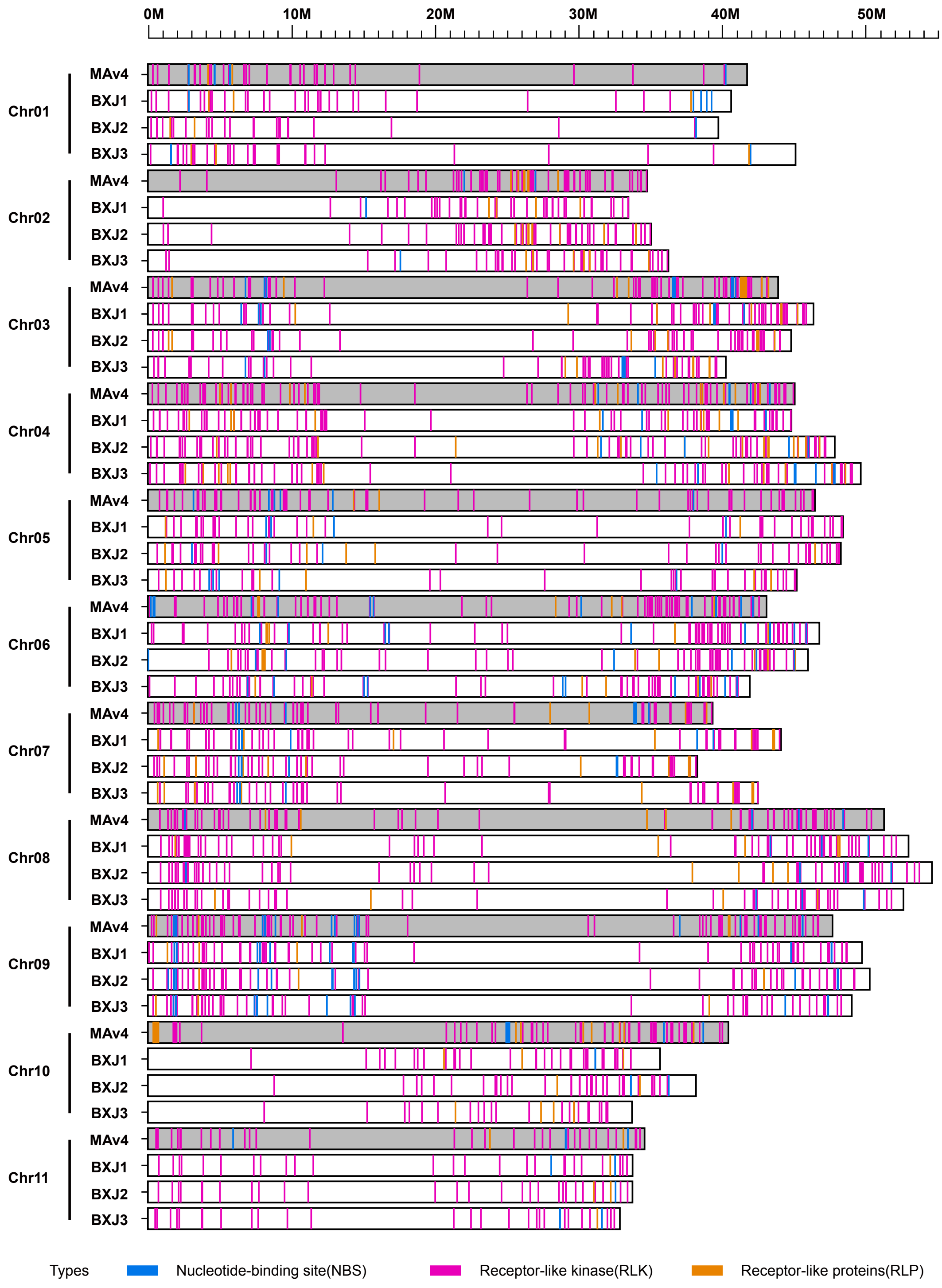

Figure S7. The distribution of the predicted RGAs in MAv4 and 'Baxijiao' genomes.

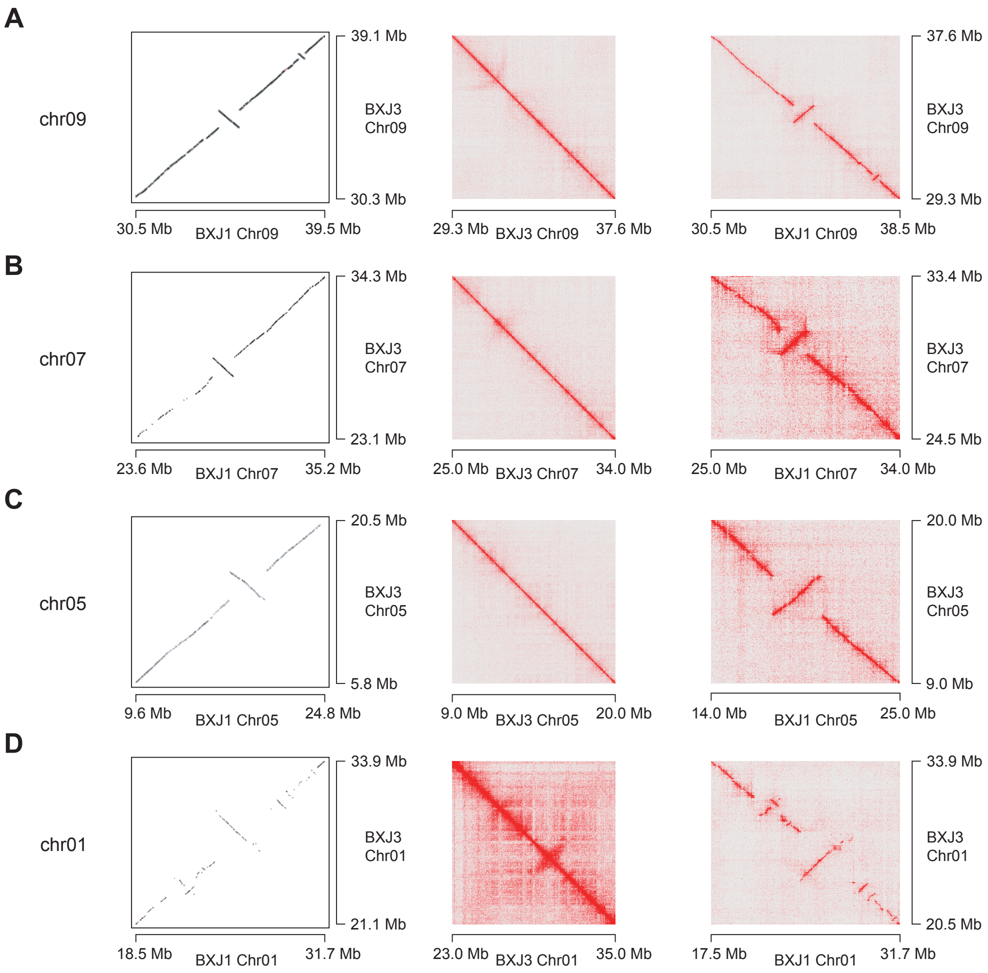

Figure S8. The Hi-C validated inversions ranging from 1.0 Mb to 3.3 Mb in chr01 (A), chr05 (B), chr07 (C), and chr09 (D). BXJ3 was used as reference genome. Hi-C contact maps were visualized at 25-kb resolution.

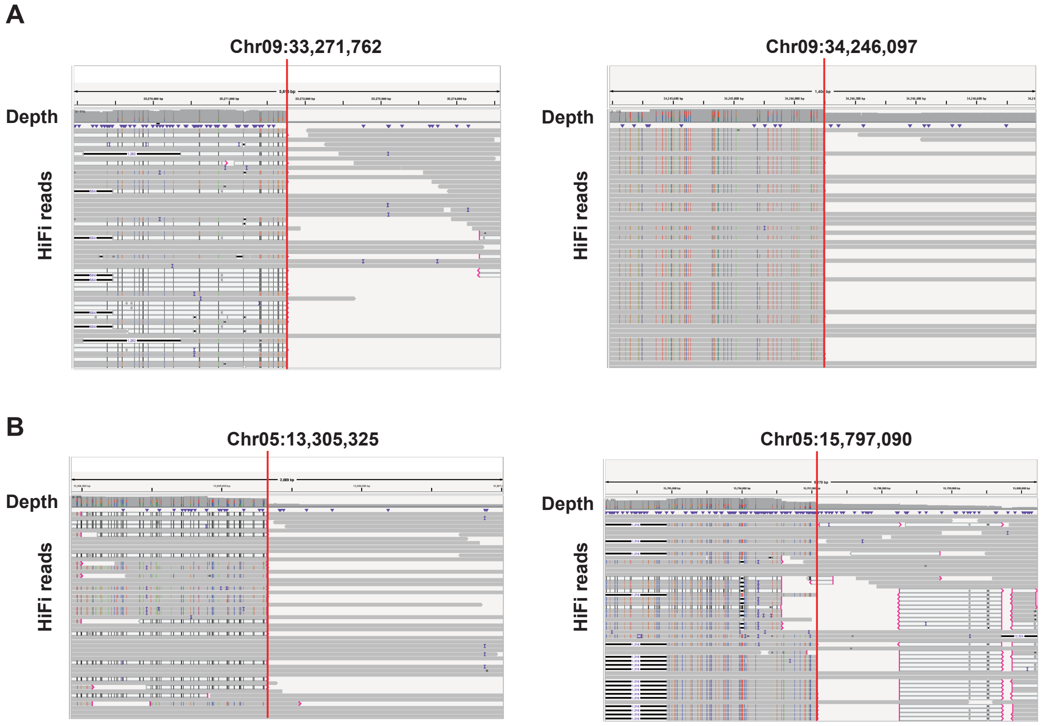

Figure S9. The Integrated Genome Viewer views of alignments to BXJ3 with HiFi reads of a 1.0 Mb inversion (Chr09: 33,271,762bp-34,246,097bp) (A) and a 2.5 Mb inversion (Chr05: 13,305,325bp-15,797,090bp) (B). Both the read depth and heterozygosity (vertical colored lines in the grey coverage plots) support that the inverted region is in a hemizygous state. Breakpoints on both sides locate in TEs.

Table S1. Summary of sequencing data generated in this study.

Types	Clean base (Gb)	Clean reads	N50 length	Depth (X) *	Sample	Application
HiFi	102.06	6,265,683	15,602	204	Leaf	Assembly
HiC	156.14	1,040,947,874	\	312.28	Leaf	Chromosome construction
ONT	48.00	1,070,015	80,356	96	Leaf	Gap filling
Illumina	99.95	\	\	199.9	Leaf	Genome evaluation
RNA-seq	6.5	\	\	\	Flower	Gene annotation and expression analysis
RNA-seq	6.3	\	\	\	Fruit	
RNA-seq	6.1	\	\	\	Leaf	
RNA-seq	6.8	\	\	\	Root	

* Sequencing depth was calculated based on average haploid genome size

Table S2. Summary of chromosome lengths of the BXJ genome.

Choromosome	Length (bp)	Mean HiFi coverage	Nb. of genes	Nb. of Contigs
BXJ1-1	40,658,927	193	3438	8
BXJ1-2	33,518,047	193.01	2438	3
BXJ1-3	46,407,064	188.23	3314	4
BXJ1-4	44,892,720	197.6	3967	5
BXJ1-5	48,464,312	197.85	3122	5
BXJ1-6	46,811,231	188.67	3892	5
BXJ1-7	44,126,930	193.77	2940	6
BXJ1-8	53,001,012	183.88	5626	1
BXJ1-9	49,765,027	201.02	3091	1
BXJ1-10	35,737,682	266.74	2880	5
BXJ1-11	33,779,584	198.3	2477	1
BXJ2-1	39,783,028	198.93	2212	4
BXJ2-2	35,110,748	189.25	2684	4
BXJ2-3	44,834,321	201.51	3691	4
BXJ2-4	47,880,837	203.38	4742	2
BXJ2-5	48,298,430	186	3435	17
BXJ2-6	46,003,104	197.19	4235	4
BXJ2-7	38,300,983	225.04	3273	7
BXJ2-8	54,632,377	180.42	3772	7
BXJ2-9	50,309,141	180.23	3384	12
BXJ2-10	38,242,695	216.09	3105	14
BXJ2-11	33,786,234	207.86	2708	7
BXJ3-1	45,134,917	184.49	3046	3
BXJ3-2	36,291,041	191.11	2537	7
BXJ3-3	40,300,839	187.94	3568	5
BXJ3-4	49,676,211	187.5	4308	4
BXJ3-5	45,251,300	183.17	2781	5
BXJ3-6	41,997,800	201.25	4149	6
BXJ3-7	42,520,259	186.1	3099	4
BXJ3-8	52,652,009	192.05	5452	6
BXJ3-9	49,064,916	205.07	3212	6
BXJ3-10	33,781,357	265.83	2556	2
BXJ3-11	32,898,040	189.2	2470	3
Total	1,423,913,123	\	111604	177

Table S3. Summary of repeat contents in BXJ1, BXJ2 and BXJ3.

**Genome name:**			**BXJ1**		
Chromosomes number:			11		
Total length (bp):			477,129,359		
GC level:			39.01%		
Bases masked:			62846558 bp (55.09%)		
			Number of elements	Length occupied (bp)	Percentage of sequence (%)
Class I: Retroelements			156,137	181,962,353	38.14
	SINEs		646	364,473	0.08
	LINEs		13,078	8,110,492	1.70
		RTE/Bov-B	7,829	2,046,031	0.43
		L1/CIN4	5,249	6,064,461	1.27
	LTR elements		142,413	173,487,388	36.36
		BEL/Pao	49	13,988	0.00
		Ty1/Copia	99,764	123,720,278	25.93
		Gypsy/DIRS1	40,520	47,631,004	9.98
		Retroviral	1,772	1,230,893	0.26
Class II: DNA transposons			5,654	1,968,058	0.41
	hobo-Activator		2,043	1,357,578	0.28
	Tc1-IS630-Pogo		110	30,879	0.01
Rolling-circles			178	175,644	0.04
Unclassified			206,233	72,552,110	15.21
Total interspersed repeats				256,482,521	53.76
Small RNA			962	388,593	0.08
Simple repeats			99,224	5,436,810	1.14
Low complexity			13,662	687,915	0.14

Genome name:			BXJ2		
Chromosomes number:			11		
Total length (bp):			477,111,996		
GC level:			39.08%		
Bases masked:			266346763 bp (55.82 %)		
			Number of elements	Length occupied (bp)	Percentage of sequence (%)
Class I: Retroelements			163,129	177,257,480	37.15
	SINEs		758	155,207	0.03
	LINEs		13,040	8,027,503	1.68
		L2/CR1/Rex	1,080	314,299	0.07
		RTE/Bov-B	6,435	1,789,022	0.37
		L1/CIN4	5,525	5,924,182	1.24
	LTR elements		149,331	169,074,770	35.44
		Ty1/Copia	100,606	119,339,801	25.01
		Gypsy/DIRS1	39,934	44,734,894	9.38
		Retroviral	102	136,477	0.03
Class II: DNA transposons			6,013	2,336,699	0.49
	hobo-Activator		3,018	1,560,830	0.33
	Tc1-IS630-Pogo		298	48,940	0.01
Rolling-circles			3,101	1,794,125	0.38
Unclassified			200,251	78,693,209	16.49
Total interspersed repeats				258,287,388	54.14
Small RNA			894	242,308	0.05
Simple repeats			98,657	5,485,853	1.15
Low complexity			13,426	674,342	0.14

**Genome name:**			**BXJ3**		
Chromosomes number:			11		
Total length (bp):			469,499,731		
GC level:			39.01%		
Bases masked:			265408669 bp (56.53 %)		
			Number of elements	Length occupied (bp)	Percentage of sequence (%)
Class I: Retroelements			144,418	165,093,979	35.16
	SINEs		1,645	527,715	0.11
	LINEs		9,835	7,160,225	1.53
		RTE/Bov-B	5,542	1,649,804	0.35
		L1/CIN4	4,293	5,510,421	1.17
	LTR elements		132,938	157,406,039	33.53
		Ty1/Copia	101,165	116,610,518	24.84
		Gypsy/DIRS1	30,630	39,655,596	8.45
		Retroviral	727	624,219	0.13
Class II: DNA transposons			5,503	2,344,150	0.50
	hobo-Activator		1,910	1,503,480	0.32
	Tc1-IS630-Pogo		210	151,091	0.03
Rolling-circles			840	408,203	0.09
Unclassified			220,783	91,402,471	19.47
Total interspersed repeats				258840600	55.13
Small RNA			1,719	549,677	0.12
Simple repeats			94,713	5,261,404	1.12
Low complexity			12,905	655,251	0.14

Table S4. Summary of Telomere information of the BXJ genome.

Chromosome	Left Start	Left End	Left Length	Right Start	Right End	Right Length
BXJ1-1	1	6,006	6,006	40,628,000	40,651,793	23,793
BXJ1-2	NA	NA	NA	33,497,737	33,515,160	17,423
BXJ1-3	1	13,951	13,951	46,383,134	46,402,615	19,481
BXJ1-4	1	13,160	13,160	44,872,247	44,886,660	14,413
BXJ1-5	1	14,273	14,273	48,415,766	48,449,604	33,838
BXJ1-6	1	21,378	21,378	46,798,668	46,807,145	8,477
BXJ1-7	1	25,452	25,452	44,096,878	44,121,539	24,661
BXJ1-8	1	16,338	16,338	52,990,441	53,000,822	10,381
BXJ1-9	1	11,774	11,774	49,732,627	49,764,071	31,444
BXJ1-10	NA	NA	NA	35,708,463	35,733,593	25,130
BXJ1-11	1	13,097	13,097	NA	NA	NA
BXJ2-1	NA	NA	NA	39,760,539	39,780,923	20,384
BXJ2-2	NA	NA	NA	35,054,222	35,072,835	18,613
BXJ2-3	1	17,213	17,213	44,813,398	44,830,275	16,877
BXJ2-4	1	14,812	14,812	47,857,243	47,869,458	12,215
BXJ2-5	NA	NA	NA	48,255,613	48,288,849	33,236
BXJ2-6	NA	NA	NA	45,970,015	45,996,223	26,208
BXJ2-7	1	14,301	14,301	38,273,305	38,297,686	24,381
BXJ2-8	1	10,514	10,514	54,620,097	54,622,442	2,345
BXJ2-9	1	15,365	15,365	50,269,023	50,304,044	35,021
BXJ2-10	NA	NA	NA	38,196,683	38,241,588	44,905
BXJ2-11	1	7,161	7,161	33,742,646	33,774,433	31,787
BXJ3-1	NA	NA	NA	45,122,259	45,129,084	6,825
BXJ3-2	NA	NA	NA	36,262,086	36,280,286	18,200
BXJ3-3	NA	NA	NA	40,270,825	40,297,789	26,964
BXJ3-4	1	11,165	11,165	49,661,570	49,673,673	12,103
BXJ3-5	1	4,494	4,494	45,226,832	45,235,253	8,421
BXJ3-6	1	16,268	16,268	NA	NA	NA
BXJ3-7	1	12,278	12,278	42,501,970	42,513,303	11,333
BXJ3-8	1	8,862	8,862	52,636,185	52,647,448	11,263
BXJ3-9	1	17,052	17,052	49,037,772	49,053,851	16,079
BXJ3-10	NA	NA	NA	33,752,348	33,768,280	15,932
BXJ3-11	1	6,090	6,090	32,879,875	32,891,936	12,061

Table S5. Summary of Centromere information of the BXJ genome.

**chromosome**	**start**	**end**	**length(bp)**	**start_trf_id**	**end_trf_id**
BXJ1-1	27,404,662	28,359,257	954,595	TRF_10123	TRF_10894
BXJ1-2	7,380,340	9,624,876	2,244,536	TRF_20324	TRF_21927
BXJ1-3	22,280,272	23,807,761	1,527,489	TRF_41249	TRF_42237
BXJ1-4	18,023,136	19,762,634	1,739,498	TRF_58991	TRF_60001
BXJ1-5	25,299,518	27,125,256	1,825,738	TRF_81060	TRF_82497
BXJ1-6	26,176,284	28,232,459	2,056,175	TRF_102206	TRF_103353
BXJ1-7	24,216,930	27,258,192	3,041,262	TRF_122036	TRF_124262
BXJ1-8	24,775,651	27,274,270	2,498,619	TRF_141710	TRF_143147
BXJ1-9	28,260,929	29,185,136	924,207	TRF_164657	TRF_165174
BXJ1-10	7,553,526	10,968,965	3,415,439	TRF_178028	TRF_179912
BXJ1-11	14,980,828	18,180,616	3,199,788	TRF_194182	TRF_196251
BXJ2-1	25,928,018	26,858,610	930,592	TRF_212670	TRF_213278
BXJ2-2	8,730,884	9,682,889	952,005	TRF_223640	TRF_224362
BXJ2-3	21,857,570	24,255,211	2,397,641	TRF_242145	TRF_243478
BXJ2-4	20,400,463	22,212,213	1,811,750	TRF_259945	TRF_260919
BXJ2-5	23,726,742	27,571,074	3,844,332	TRF_279591	TRF_282151
BXJ2-6	25,957,054	27,320,570	1,363,516	TRF_301401	TRF_302160
BXJ2-7	17,005,447	20,720,923	3,715,476	TRF_315107	TRF_317728
BXJ2-8	24,022,288	27,846,240	3,823,952	TRF_336147	TRF_338591
BXJ2-9	30,094,891	31,095,968	1,001,077	TRF_365209	TRF_365652
BXJ2-10	9,798,246	11,748,382	1,950,136	TRF_379774	TRF_380822
BXJ2-11	15,547,471	17,858,582	2,311,111	TRF_396817	TRF_398179
BXJ3-1	26,268,760	27,208,758	939,998	TRF_414500	TRF_415083
BXJ3-2	9,645,348	11,264,148	1,618,800	TRF_427570	TRF_428608
BXJ3-3	20,776,674	21,080,707	304,033	TRF_447868	TRF_448056
BXJ3-4	15,825,103	19,905,567	4,080,464	TRF_462189	TRF_465175
BXJ3-5	21,631,806	22,993,250	1,361,444	TRF_488366	TRF_489398
BXJ3-6	22,732,558	23,223,747	491,189	TRF_508081	TRF_508339
BXJ3-7	23,668,002	25,778,531	2,110,529	TRF_526970	TRF_528467
BXJ3-8	20,894,102	22,793,191	1,899,089	TRF_544247	TRF_545293
BXJ3-9	27,504,384	28,958,483	1,454,099	TRF_569963	TRF_570895
BXJ3-10	8,815,954	9,310,419	494,465	TRF_584467	TRF_584664
BXJ3-11	17,184,607	17,666,240	481,633	TRF_600740	TRF_601104

Table S9. Statistics of the significantly expanded and contracted gene families.

	**Expansion families (OGs)**	**Expansion genes**	**Contraction families (OGs)**	**Contraction genes**
Baxijiao1(BXJ1)	210	1574	339	867
Baxijiao2(BXJ2)	251	1665	360	903
Baxijiao3(BXJ3)	183	1238	460	1390
Musa acuminata	510	3693	193	414
Musa balbisiana	207	1745	683	1990
Musa schizocarpa	279	1821	507	1289
Musa beccarii	416	2840	254	701
Musa troglodytarum	359	2548	408	1403

Table S17. Species pairs and their estimated divergence times used for time calibration points to infer time-calibrated phylogeny.

**Species pair**	**Estimated divergent time (million years ago, MYA)**
Oryza sativa-Zea mays	41.4-51.9
Oryza sativa-Ananas comosus	94.1-117
Musa acuminata - Zingiber officinale	50.6-91.1
Zingiber officinale - Elaeis guineensis	103.0-119.6
Asparagus officinalis - Elaeis guineensis	108.4-123.6
Zingiber officinale- Ananas comosus	103.2-117.1
Spirodela intermedia- Asparagus officinalis	125.3-137.9
Zea mays - Setaria viridis	17.5-29.5
Musa acuminata - Ensete_glaucum	45.1-69.1

Note: this is from www.timetree.org website.

Table S18. The thresholds of the relative expression levels for each homoeolog expression bias category.

**Category**	**BXJ1**	**BXJ2**	**BXJ3**
Balanced	0.2-0.8	0.2-0.8	0.2-0.8
BXJ1 Dominant	0.8-1.0	0-0.2	0-0.2
BXJ2 Dominant	0-0.2	0.8-1.0	0-0.2
BXJ3 Dominant	0-0.2	0-0.2	0.8-1.0
BXJ1 Suppressed	0-0.2	0.2-0.8	0.2-0.8
BXJ2 Suppressed	0.2-0.8	0-0.2	0.2-0.8
BXJ3 Suppressed	0.2-0.8	0.2-0.8	0-0.2

Note: BXJ1, BXJ2 and BXJ3 represent the relative expression levels of the BXJ1, BXJ2 and BXJ3 homoeologs across an individual triad. The relative expression levels of a homoeolog were estimated as follows: expression_{BXJ1} = TPM(*BXJ1*) / (TPM(*BXJ1*) + TPM(*BXJ2*) + TPM(*BXJ3*)), expression_{BXJ2} = TPM(*BXJ2*) / (TPM(*BXJ1*) + TPM(*BXJ2*) + TPM(*BXJ3*)), expression_{BXJ3} = TPM(*BXJ3*) / (TPM(*BXJ1*) + TPM(*BXJ2*) + TPM(*BXJ3*)).
